# Protein Kinase C Epsilon Overexpression Is Associated With Poor Patient Outcomes in AML and Promotes Daunorubicin Resistance Through p-Glycoprotein-Mediated Drug Efflux

**DOI:** 10.3389/fonc.2022.840046

**Published:** 2022-05-30

**Authors:** Rachael Nicholson, Ana Catarina Menezes, Aleksandra Azevedo, Adam Leckenby, Sara Davies, Claire Seedhouse, Amanda Gilkes, Steve Knapper, Alex Tonks, Richard L. Darley

**Affiliations:** ^1^ Department of Haematology, Division of Cancer and Genetics, School of Medicine, Cardiff University, Cardiff, United Kingdom; ^2^ Academic Haematology, Nottingham University Hospitals and University of Nottingham, Nottingham, United Kingdom; ^3^ Cardiff Experimental and Cancer Medicine Centre (ECMC), School of Medicine, Cardiff University, Cardiff, United Kingdom

**Keywords:** protein kinase C epsilon, acute myeloid leukemia, daunorubicin, drug resistance, P-glycoprotein (ABCB1 protein)

## Abstract

The protein kinase C (PKC) family of serine/threonine kinases are pleiotropic signaling regulators and are implicated in hematopoietic signaling and development. Only one isoform however, PKCϵ, has oncogenic properties in solid cancers where it is associated with poor outcomes. Here we show that PKCϵ protein is significantly overexpressed in acute myeloid leukemia (AML; 37% of patients). In addition, PKCϵ expression in AML was associated with a significant reduction in complete remission induction and disease-free survival. Examination of the functional consequences of PKCϵ overexpression in normal human hematopoiesis, showed that PKCϵ promotes myeloid differentiation, particularly of the monocytic lineage, and decreased colony formation, suggesting that PKCϵ does not act as an oncogene in hematopoietic cells. Rather, in AML cell lines, PKCϵ overexpression selectively conferred resistance to the chemotherapeutic agent, daunorubicin, by reducing intracellular concentrations of this agent. Mechanistic analysis showed that PKCϵ promoted the expression of the efflux pump, P-GP (ABCB1), and that drug efflux mediated by this transporter fully accounted for the daunorubicin resistance associated with PKCϵ overexpression. Analysis of AML patient samples also showed a link between PKCϵ and P-GP protein expression suggesting that PKCϵ expression drives treatment resistance in AML by upregulating P-GP expression.

## Introduction

Acute myeloid leukemia (AML) describes an aggressive group of hematological malignancies characterized by the accumulation of clonal, abnormally differentiated myeloid cells in the bone marrow (BM) and peripheral blood. For most patients, prognosis remains poor using conventional chemotherapeutic strategies, with an average survival of <6 months (1). Consequently, understanding the molecular mechanisms which underpin this malignancy with the aim of developing targeted therapeutic approaches to improve patient outcomes is a central focus in this field.

The PKC family of serine/threonine kinases consists of 11 isoforms which are classified according to their structure, cofactor activation and substrate specificity into classical (cPKC; α, βI/II, γ), novel (nPKC; δ, ϵ, η, θ) and atypical (aPKC; ζ, λ/ι) isoforms (2). Of these, the nPKC isoform PKCϵ is unique within its family having shown transforming oncogenic properties and is frequently upregulated in solid cancers, where it is associated with aggressive disease phenotypes (3–6). In a hematological context, PKCϵ promotes erythrocyte and megakaryocyte lineage commitment and differentiation (7, 8). PKCϵ has also been implicated in malignant cell survival (9–12) and in AML cells PKCϵ has been reported to protect from TRAIL-induced apoptosis and to modulate reactive oxygen species (ROS) homeostasis (13, 14). Here we examine the frequency of PKCϵ dysregulation, as well as the pathophysiological associations and mechanistic contribution of PKCϵ misexpression in AML.

P-glycoprotein (P-GP) is an ATPase efflux pump from the ATP-binding cassette (ABC) transporter group. Substrates for ABC efflux pumps such as P-GP include amino acids, organic ions, peptides, chemotherapeutic agents, and xenobiotics (15). In AML, P-GP overexpression is associated with poor outcomes in newly diagnosed and relapsed disease (16). Particularly pertinent in AML is the ability of pumps such as P-GP to confer resistance to anthracyclines but has also been linked to increased invasiveness of AML cell lines (17). The mechanisms regulating efflux pump expression and activity are highly complex and have not been fully resolved. Despite this, some aspects of efflux pump regulation are outlined below, with a focus on P-GP, as this is the best characterized drug transporter. A wide range of transcription factors including AP-1 and NF-κB have been implicated in P-GP regulation (18, 19). The regulation of P-GP is post-translational as well as transcriptional. P-GP is exported from the endoplasmic reticulum as a 150-kDa protein which is subsequently glycosylated in the Golgi apparatus and yields the mature 170-kDa protein which mediates drug efflux (20). In addition to expression, the activity of efflux pumps is also central to drug transport. A role for PKC activity in activating P-GP have primarily been established using PKC agonists and inhibitors (21, 22). Three PKC phosphorylation residues (ser661, Ser667 and Ser 671) have been identified within the intracellular linker region of the P-GP peptide, suggesting potential roles for this family of kinases in P-GP regulation (23); however, directed mutagenesis of the PKC phosphorylation sites did not affect P-GP expression or activity in a yeast model (24). PMA treatment has been shown to promote P-GP expression and drug efflux (23, 25) while inhibiting PKC isoforms can overcome these phenotypes (26). However, the causality of these findings is complicated by the fact that some PKC inhibitors, including Chelerythrine and Enzastaurin can suppress P-GP-mediated drug resistance by directly binding P-GP and inhibiting its function (23, 27, 28).

## Material and Methods

### Primary Cell Material and Cell Culture

Diagnostic peripheral blood (PB) and BM samples were from patients who went on to receive intensive chemotherapy within the AML14 and AML15 NCRI trials (**Supplementary Table 1**). Cord blood was obtained from the University Hospital of Wales or NHS Blood and Transplant (NHSBT). All sample collections were in accordance with the 1964 Declaration of Helsinki. AML cell lines were purchased from ATCC and ECACC and were maintained as recommended. Cell line identity was confirmed using Cell Line Authentication Service (February 2021; Eurofins Scientific, Luxembourg). CD34^+^ hematopoietic stem/progenitor cells (HSPC) were isolated, cultured, and transduced with lentivirus as previously described (29). PKCϵ overexpression in HSPC and AML cell lines, employed a lentiviral expression construct, PKCϵ_GFP-Puro, expressing the *PRKCE* gene (NM0-05400.3) under the control of the *EF1A* promoter and *EGFP* and *puro* ^R^ genes linked by a T2A sequence driven by a CMV promoter. Control cells were transduced with vector lacking the *PRKCE* gene. Knockdown of PRKCE was achieved with shRNA lentiviral vectors (detailed in **Supplemental Methods**). Control cells were transduced with a non-targeted construct. All vectors were purchased from VectorBuilder Inc (Chicago, USA). Transduced AML cell lines were selected with puromycin at 1µg/ml.

Myeloid colony forming assays were performed by limiting dilution in U bottomed 96-well plates (0.3 cells/well) using transduced (GFP^+^) HSPC enriched for cells highly expressing CD34 (CD34^+^) by FACS on day 3 of culture. Cells were cultured in Iscove’s Modified Dulbecco’s Medium (IMDM; Fisher Scientific, Loughborough, UK) supplemented with 5 ng/mL IL-3, SCF, G-CSF and GM-CSF and incubated at 37°C with 5% CO_2_. Following 7 days of growth, individual colonies (> 50 cells) and clusters (> 5, < 50 cells) were scored.

### Western Blot Analysis

Western blot analysis was conducted as previously described (30) using the antibodies described in **Supplementary Table 2**. Prior to western blot and colony analysis of transduced HSPC (GFP^+^) cells were purified by FACS using AriaII (BD Biosciences). Band intensity on documented membranes were measured by densitometric analysis using ImageJ (Fiji; v. 2.0.0.71), unless otherwise stated. To do this, a region of interest (ROI) was constructed around a specific band. From this, a histogram of peak intensity was generated, and a baseline of background intensity was set from the area surrounding the band within the ROI. The area under the curve was then calculated to give an arbitrary intensity value. The band intensities of the protein for each sample were normalized to the band intensity of the loading control (GAPDH expression). PKCϵ expression in AML patient samples were subsequently converted into fg/1000 cells by comparing the relative band intensity to a recombinant PKCϵ standard of a known concentration.

### Analysis of *PKCϵ* Expression

*PKCϵ* (*PRKCE*) mRNA expression data from human (MicroArray; GSE42519 (31);) and murine (GSE14833 (32); and GSE6506 (33, 34)) HSPC were obtained from Bloodspot (35). For AML patient samples, *PKCϵ* mRNA expression was assessed using the TCGA, NEJM 2013 dataset (36) which was accessed using cBioPortal (37, 38). For survival analysis, only patients that received intensive cytarabine (Ara-C) and daunorubicin (DNR) treatment regimens were included. Patients were subsequently stratified according to *PKCϵ* expression using the upper (high *PKCϵ*) and lower (low *PKCϵ*) quartiles. *PKCϵ* (*PRKCE*) mRNA expression was also determined in AML14 and AML15 NCRI UK clinical trial samples by Affymetrix DNA microarray. The raw data from the Hu133A GeneChip^®^ was normalized using MAS5 or Robust Multi Array analysis. Two probe-sets were used (236459_at and 226101_at) as these had the highest correlation and the highest association with MAS5 present calls (20/22). The DEPMAP portal was used to analyze transcriptional correlations in 44 AML cell lines using the Expression 22Q1 Public RNASeq data set.

### Flow Cytometric Analysis of Drug Sensitivity, Immunophenotype, and Cell Cycle

Flow cytometric data were acquired using an Accuri C6 cytometer (BD Biosciences, USA) and analyzed using FCS Express v6 (DeNovo, California, USA). For cell growth, viability, and drug sensitivity assays, cells were seeded at 2x10^5^/mL. After 48 hours, harvested cells were stained with a TOPRO-3 staining solution (RPMI supplemented with 100 mM HEPES and 50nM TOPRO-3 (T3605; Invitrogen, California, USA). For flow cytometric analysis, debris acquired during sample acquisition, defined as TOPRO-3 negative events with a FSC of <5x10^4^, were excluded and viability and viable cell counts were determined from within the viable cell population of TOPRO-3 negative events. Absolute counts/mL were determined from a 10 µL fixed volume acquisition. For the drug sensitivity assays, cell growth and viability were normalized to cells treated with the vehicle control. Drug sensitivity was compared by calculating the drug concentration which gave a half-maximal response (EC50). Master stocks of each agent were generated according to the manufacturer’s instructions and are outlined in **Supplementary Table 3**. Once generated, all stocks were stored at -20°C in 100 µL aliquots for long-term storage unless otherwise stated. DNR accumulation was determined by measuring the arithmetic mean fluorescence (585/40nm) for cells treated with 100nM DNR, compared to vehicle controls. Immunophenotypic analysis was performed as previously described (39). P-glycoprotein (P-GP) expression on transduced AML cell lines was determined using the CD243-APC antibody (UIC2; BioLegend; **Supplementary Table 2**), while expression in patient samples has been described previously (40). Full details of the all the antibodies used for immunophenotypic studies are outlined in **Supplementary Table 2**.

For cell cycle analysis, transduced AML cell lines were seeded at 2x10^5^ cells/mL and expanded for 48 hours before being harvested to 1 mL flow tubes and washed with 1 mL PBS. The washed cells were subsequently resuspended in PBS and fixed for 30 min on ice by adding 700 µL absolute ethanol. After fixation, the tubes were stored at -20°C overnight. The next day, the cells were centrifuged at 270*xg* and washed with 1 mL PBS before being resuspended in 50 µL staining buffer (PBS+0.5% (*w/v*) BSA+ 0.02% (*w/v*) sodium azide). The cells were then stained with 25 µL staining solution containing 40 µg/mL propidium iodide, and 0.1 mg/mL RNase (Sigma-Aldrich) diluted in PBS, for 30 min at 37°C. Samples were acquired within 20 min of this incubation using the Accuri™ C6 Plus flow cytometer. For analysis, debris and doublets were excluded, before the cell cycle status was resolved using the fluorescence intensity of propidium iodide (Sigma-Aldrich). Cell cycle analysis was performed using the Multicycle AV DNA analysis tool plug-in for FCS Express (DeNovo, Pasadena, USA).

### Statistical Analysis

Statistical significance was determined by the tests outlined in figure legends and were performed using GraphPad Prism v 9.0.

## Results

### PKCϵ Is Frequently Upregulated in AML and Is Associated With Poor Clinical Outcome

**Figure 1:**
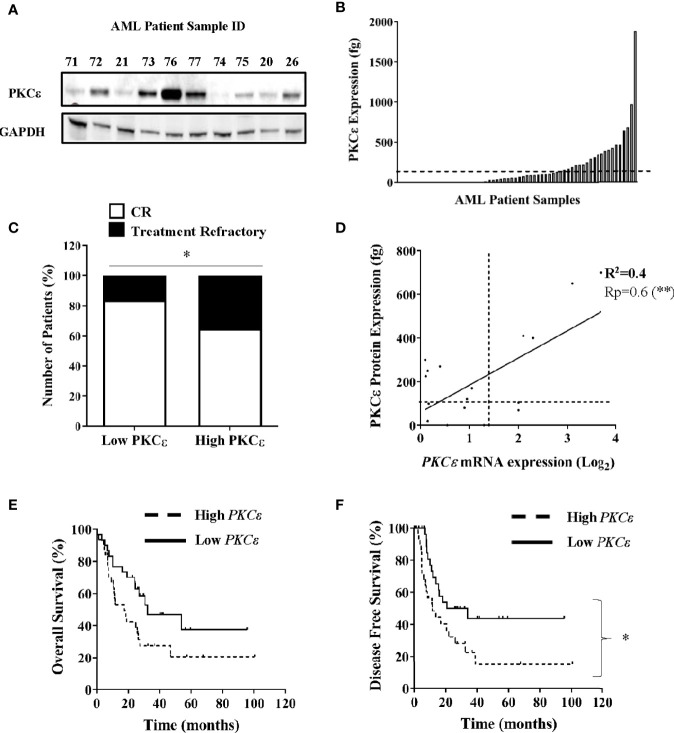
PKCϵ is frequently overexpressed in AML and is associated with poor patient outcomes. **(A)** Example western blot showing PKCϵ (MW-84kDa) protein expression in AML patient samples from the AML14 and AML15 trials (**Supplementary Table 1**). PKCϵ expression was detected using the C-15 Santa Cruz antibody (**Supplementary Table 2**) and is shown alongside GAPDH (MW-36kDa) which was used as a loading control (6c5 antibody; Santa Cruz; **Supplementary Table 2**). **(B)** Bar chart showing PKCϵ protein expression in AML patients (fg protein/1000 cells; n=70). PKCϵ expression was quantified by western blot. Dotted line shows threshold for overexpression [>2SD from mean of normal CD34^+^ BM blasts (n=4)]. **(C)** Proportion of patients achieving CR vs refractory disease following induction chemotherapy for patients with high (overexpressing) and low PKCϵ (<threshold for overexpression); *p < 0.05 using Fisher’s Exact test. **(D)** Correlation between *PKCϵ* mRNA expression (Log_2_), determined by Hu133A GeneChip^®^ microarray analysis and protein expression (as A), in AML patients (n=18). Dotted lines show threshold for mRNA and protein overexpression compared to normal CD34^+^ BM blasts, Pearson’s correlation analysis (Rp) 0.6; 95% CI (0.24-0.85); **p < 0.01. **(E)** Impact of PKCϵ expression on OS and **(F)** Impact on DFS for AML patients from the TCGA dataset (36), where high (n=31) and low (n=30) *PKCϵ* expression were defined as the upper and lower quartiles respectively [High *PKCϵ*: median survival- 17.4 months vs low *PKCϵ-*32.3 months, HR 1.89 95%CI (0.99-3.61)], [High *PKCϵ*: median DFS- 11.6 months vs low *PKCϵ-*27.45 months, HR 2.1 95%CI (1.1-4.2)].

PKCϵ has demonstrated oncogenic properties and in solid cancers misexpression is associated with poor clinical outcomes and aggressive disease phenotypes (3–5, 41). To elucidate the frequency and pathophysiological attributes associated with PKCϵ upregulation in AML, we assessed PKCϵ expression in two independent patient sample cohorts. Initial data showed PKCϵ protein to be broadly expressed in AML (**Figure 1A**). We next carried out quantitative assessment in 70 AML patient samples. Compared to normal CD34^+^ BM blasts, PKCϵ protein was significantly overexpressed in 37% (26/70) of samples analyzed (**Figure 1B**). Patients overexpressing PKCϵ protein had a significantly lower complete remission (CR) rate compared to those with low PKCϵ protein expression (65% vs 84%; **Figure 1C**) with no significant association between any other prognostically significant patient characteristic. A possible explanation for this is that PKCϵ overexpression is associated with chemotherapy resistance. To validate these findings, we used a clinically annotated mRNA dataset. We first established a significant positive correlation between PKCϵ mRNA and protein expression in AML patient samples (**Figure 1D**) which indicated that mRNA is a reasonable predictor of PKCϵ protein expression in this context but found no relationship to FAB subtype or LSC phenotype (data not shown). We subsequently evaluated the impact of *PKCϵ* mRNA expression in AML patients using the TCGA dataset (36). High *PKCϵ* mRNA expression was not significantly associated with any AML subset or known prognostic factors (**Supplementary Figure 1**). While the impact on overall survival (OS) was not significant (**Figure 1E**; p=0.052), high *PKCϵ* expression was significantly associated with reduced disease-free survival (DFS; **Figure 1F**). Although CR status was not available, reduced DFS is consistent with a treatment resistant phenotype. This data therefore supports the protein analysis in suggesting that high PKCϵ expression is associated with poor outcomes in AML. Together these data show that PKCϵ is frequently upregulated in AML and indicate that high PKCϵ expression is associated with poor patient outcomes, particularly in terms of response to initial chemotherapy.

### PKCϵ Overexpression Promotes Monocytic Differentiation

**Figure 2:**
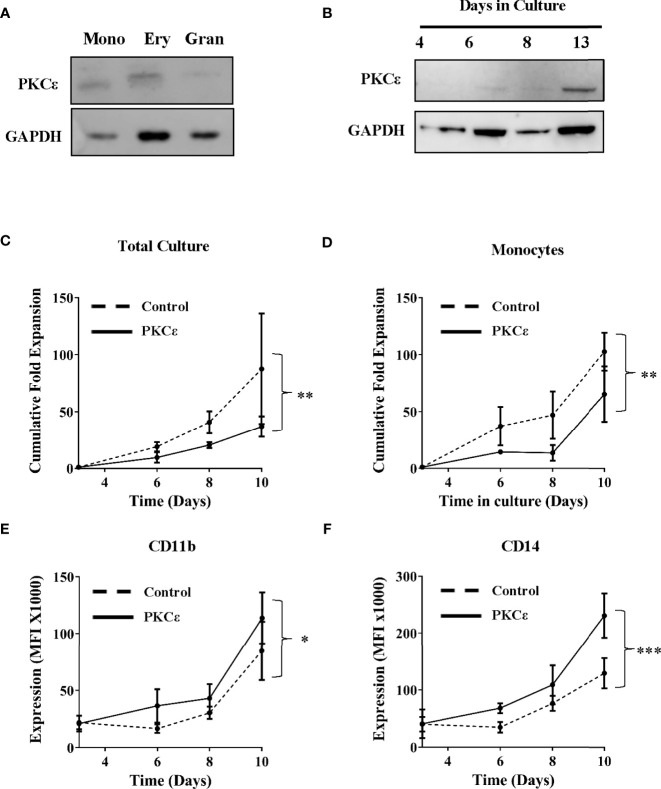
PKCϵ is expressed throughout hematopoiesis and promotes monocyte differentiation. **(A)** Western blot showing PKCϵ (MW-84kDa) protein expression in human umbilical cord blood (CD34^+^) derived monocyte (mono; CD14^high^), erythrocyte (very; CD14^neg^CD36^high^) and granulocyte (gran; CD14^low^CD36^low^) progenitors. **(B)** Western blot analysis showing PKCϵ protein (MW-84kDa) expression in CD34^+^ HSPC over 13 days of culture where PKCϵ expression is shown alongside GAPDH (MW-36kDa) expression which was used as a loading control. For densitometry analysis of this western blot see **Supplementary Figure 3A**. PKCϵ expression was detected using the Cell Signaling Technologies antibody (22B10; **Supplementary Table 2**) while GAPDH was detected using the ThermoFisher Scientific antibody (GA1R; **Supplementary Table 2**). **(C)** Cumulative fold expansion of CD34^+^ HSPC total culture and **(D)** of monocytic progenitors (CD13^high^CD36^high^) transduced with the control or PKCϵ overexpression constructs. Immunophenotypic analysis of **(E)** CD11b and **(F)** CD14 expression (MFI) on transduced monocytic progenitors (CD13^high^CD36^high^) over 10 days of culture. Antibodies used for this analysis are outlined in **Supplementary Table 2**; n=4, *p < 0.05, **p < 0.01, ***p < 0.001 using two-way ANOVA with Bonferroni post-test comparison between the control and PKCϵ overexpression cells at day 10 of culture.

Having determined that PKCϵ is frequently upregulated in AML, the mechanistic contribution of PKCϵ misexpression was evaluated. Initially we focused on whether overexpression contributes to the pathogenesis of AML by disrupting myeloid cell growth and differentiation. We first examined *PKCϵ* mRNA expression throughout normal myeloid development. In human and murine myeloid progenitors, *PKCϵ* was highly expressed in HSC and MPP, however expression was observed throughout hematopoiesis (**Supplementary Figure 2**), indicating that *PKCϵ* may influence myeloid lineage commitment and differentiation. To support this, western blot analysis of human HSPC confirmed PKCϵ expression in all myeloid progenitor subsets (**Figure 2A**). The migratory differences observed are attributable to different phosphorylation states of PKCϵ (42). Further, when HSPC were cultured in conditions primarily supporting monocytic and granulocytic differentiation, PKCϵ expression increased with maturation (**Figure 2B**). Together these data indicate a potential developmental role for PKCϵ in myelopoiesis; however, we were unable to show any developmental consequences arising from PKCϵ knockdown in this context (**Supplementary Figures 3-6**) though we did observe a significant reduction in the growth of granulocytic cells (**Supplementary Figure 4**).

We next examined the functional consequences of PKCϵ overexpression. CD34^+^ HSPC stably overexpressing PKCϵ (**Supplementary Figures 7B-C**) were analyzed for changes in growth and differentiation by flow cytometry. Briefly, the myeloid lineages were resolved using the lineage discrimination markers CD13 and CD36, into monocytic (CD13^high^CD36^high^) and granulocytic (CD13^low^CD36^low)^ progenitor populations (**Supplementary Figure 8**). Overall PKCϵ overexpression was associated with a 2.6-fold reduction in HSPC fold expansion over 10 days of culture (**Figure 2C**) and had a similar impact on colony formation (**Supplementary Figure 9**). Subset analysis revealed that PKCϵ overexpression significantly reduced the growth of monocytic progenitors (1.6-fold), compared to the control cultures (**Figure 2D**). This was associated with significant upregulation of the maturation markers CD11b (**Figure 2E**) and CD14 (**Figure 2F**). In contrast, we observed no significant impact on granulocytic proliferation (**Supplementary Figure 10A**) or differentiation (**Supplementary Figures 10B, C**). Together, these data indicate that increased PKCϵ expression promotes monocyte differentiation and does not support an oncogenic role for PKCϵ upregulation in the pathogenesis of AML, which is characterized by a block in terminal differentiation.

### PKCϵ Overexpression Promotes Selective DNR Resistance in AML Cell Lines

**Figure 3:**
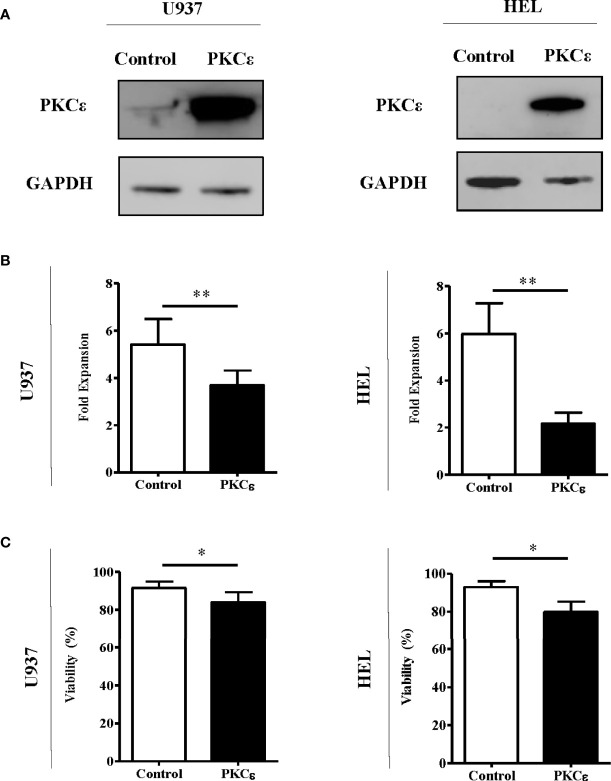
PKCϵ overexpression reduces the fold expansion of AML cell lines. **(A)** Representative western blots showing PKCϵ (MW-84kDa) expression in U937 and HEL cells transduced with the control or PKCϵ overexpression constructs. PKCϵ expression is shown alongside GAPDH (MW-36kDa) expression which was used as a loading control; n=2. PKCϵ expression was detected using the Cell Signaling Technologies antibody (22B10; **Supplementary Table 2**) while GAPDH was detected using the ThermoFisher Scientific antibody (GA1R; **Supplementary Table 2**). **(B)** Fold expansion and **(C)** viability of U937 and HEL cells transduced with the control or PKCϵ overexpression constructs 48 hours after seeding. Fold expansion and viability were determined using TOPRO-3 staining; n=4; *p < 0.05, **p < 0.01 using paired t-test.

Given that the data above are not supportive of an oncogenic role for PKCϵ overexpression in AML, we next examined the capacity of PKCϵ to promote chemoresistance in AML cells as this is a known consequence of PKCϵ overexpression in solid cancers (10, 41). To model this, we overexpressed PKCϵ in AML cell lines (U937 and HEL) which exhibited low and undetectable levels of endogenous PKCϵ protein, respectively. Ectopic PKCϵ expression was validated by western blot analysis (**Figure 3A**) and the resulting impact on cell proliferation and viability were assessed by flow cytometry. PKCϵ overexpression resulted in a 1.5-fold (U937) and 2.8-fold (HEL) reduction in growth compared to the control lines (**Figure 3B**). This was accompanied by a modest increase in the proportion of cells in the G2 phase of the cell cycle (**Supplementary Figure 11**), suggesting that the reduced proliferation rate could be due to perturbed cell cycle progression. Although PKCϵ overexpression also resulted in a 10% reduction in cell viability (**Figure 3C**), the small effect size suggests this is unlikely to be biologically significant and may have arisen from the relative accumulation of non-viable cells due to the reduced proliferation rate of the PKCϵ cultures.

**Figure 4:**
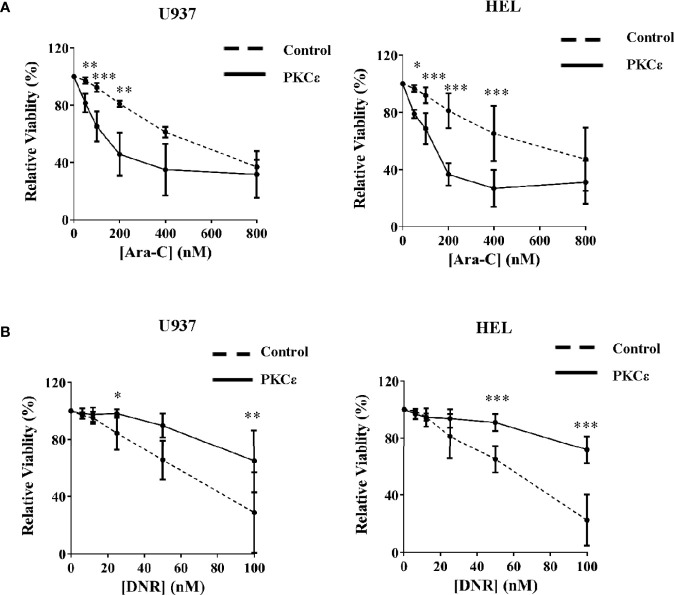
PKCϵ overexpression selectively promotes resistance to DNR. Line graphs showing the effect of increasing **(A)** Ara-C and **(B)** DNR concentrations on the viability of U937 (left) and HEL (right) cells transduced with the control or PKCϵ overexpression constructs, following 48 hours of treatment. Viability was determined by flow cytometry using TOPRO-3 staining (**Supplemental Methods**) and was normalized to the viability of cells treated with the vehicle control (PBS), at the time of harvesting; n=3; data represents mean ±1SD; *p < 0.05, **p < 0.01, ***p < 0.001 comparing test and control cultures at the dose indicated using two-way ANOVA with Bonferroni post-test comparison.

We next established the effect of PKCϵ overexpression on Ara-C and DNR chemosensitivity; two of the central agents used in AML treatment. Despite the reduced growth of these cell lines, PKCϵ overexpression significantly sensitized U937 and HEL cells to Ara-C (2.9-fold and 1.9-fold respectively; **Figure 4A**), therefore inconsistent with the hypothesis that PKCϵ overexpression confers chemoresistance to this nucleotide analogue. In contrast however, PKCϵ overexpression increased the EC50 of DNR from 60nM to >100nM in both cell lines investigated (**Figure 4B**), demonstrating that PKCϵ overexpression can selectively confer resistance to DNR.

### PKCϵ-Mediated DNR Resistance Arises From P-GP-Mediated Drug Efflux

As PKCϵ overexpression in AML cell lines promoted opposing phenotypes to Ara-C and DNR, it was initially hypothesized that the mechanism of PKCϵ-mediated chemoresistance was specific to the mode of action of DNR. The cytotoxicity of DNR is, in part, mediated by oxidative stress (43). Since PKCϵ has been reported to promote antioxidant capacity in AML (14) we assessed whether the PKCϵ overexpression lines showed resistance to other agents which induce oxidative stress (glucose oxidase, antimycin A, and arsenic trioxide). We found no evidence of resistance to any of these agents (rather sensitization was observed; **Supplementary Figure 12**) indicating that enhanced antioxidant capacity is unlikely to explain DNR resistance.

**Figure 5:**
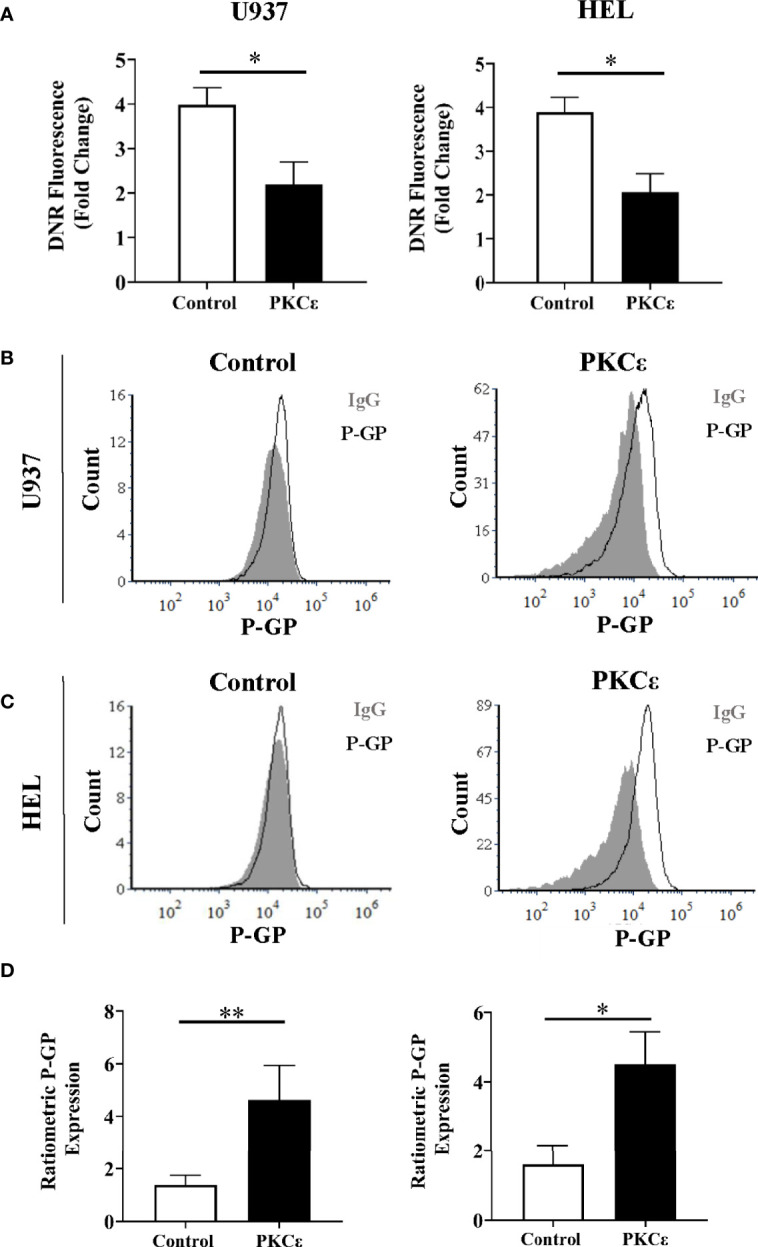
PKCϵ overexpression promotes P-GP upregulation in AML cell lines. **(A)** Bar charts showing the DNR fluorescence [fold change relative to cells treated with the vehicle control, (PBS)] of U937 (left) and HEL cells (right) transduced with the control and PKCϵ overexpression constructs, following 2 hours of treatment with 100nM DNR (n=4); data represents mean+1SD; * p<0.05 using paired t-test. **(B, C)** Representative histograms showing P-GP expression (MFI) in U937 and HEL cells transduced with the control and PKCϵ overexpression constructs. P-GP expression was detected by flow cytometry using the CD243-APC (UIC2; **Supplementary Table 2**) antibody and was compared to an isotype control (IgG2k-APC). **(D)** Corresponding bar charts showing ratiometric P-GP expression. P-GP expression was determined by flow cytometry using the CD243-APC (UIC2; **Supplementary Table 2**) antibody and was compared to an isotype control (IgG2k-APC; **Supplementary Table 2**); U937 (left) and HEL (right) (n=4), data represents mean+1SD; *p < 0.05, **p < 0.01 using paired t-test.

We next investigated whether PKCϵ influenced intracellular DNR concentrations. DNR has intrinsic fluorescence enabling direct measurement of intracellular drug accumulation by flow cytometry. Following 2 hours of DNR treatment, the PKCϵ overexpression cell lines showed a 1.8-fold reduction in intracellular DNR compared to the control lines (**Figure 5A**) with similar data observed at the timepoint used for cytotoxicity assessment (2 days of treatment; **Supplementary Figure 13A**). Reduced DNR accumulation can arise from decreased drug uptake or increased expulsion *via* efflux pumps. DNR enters the cell by passive diffusion and so is unlikely to explain the observed reduction in intracellular DNR. Instead, it was hypothesized that the reduced DNR accumulation was due to increased drug efflux. P-GP (aka ABCB1/MDR1/CD243) is the best characterized efflux pump and is associated with reduced OS and CR induction in AML (44, 45). P-GP has several substrates including DNR (46), but does not transport Ara-C (47), potentially explaining the differential phenotypes exhibited in response to these chemotherapeutic agents.

**Figure 6:**
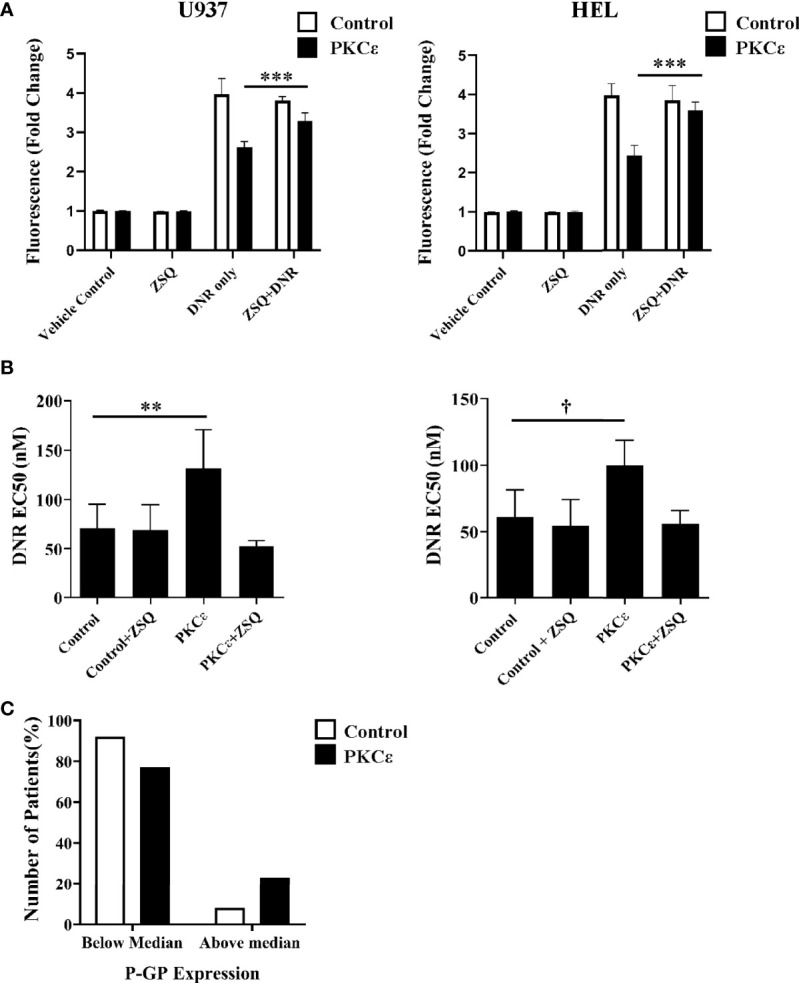
P-GP inhibition promotes DNR accumulation and sensitization in PKCϵ overexpression cell lines while high P-GP and PKCϵ protein expression is observed in AML blasts. **(A)** Bar charts showing DNR fluorescence (fold change relative to the fluorescence of cells treated with the vehicle control), following 2 hours of treatment with 100nM DNR and 100nM ZSQ in U937 (left) and HEL (right) cells. DNR and ZSQ co-treatment is shown alongside the fluorescence of cells treated with the vehicle control (PBS+0.025% (*v/v*) DMSO) or 100nM ZSQ alone (n=3), data represents mean +1SD; ***p < 0.001 using one-way ANOVA with Bonferroni post comparison test where statistical comparisons were made between the DNR alone and DNR+ZSQ treatments. No significance was observed between these treatments for the control cell lines. **(B)** Bar charts showing the effect of ZSQ treatment (100nM) on the EC50 (nM) of DNR, following 48 hours of treatment in U937 (left) and HEL (right) cells. Viability was determined by flow cytometry using TOPRO-3 staining and normalized to cells treated with the vehicle control (n=3), data represents mean+1SD; **p < 0.01, ^†^p=0.06 using one-way ANOVA with Bonferroni post-test comparison. The dose response curves for DNR alone and DNR+ZSQ treatments are available in the **Supplementary Figure 13D**. **(C)** Bar chart showing the number of patients with above or below median P-GP protein expression in AML patients overexpressing PKCϵ at the protein level (compared to normal CD34^+^ blasts; n=14) or with low PKCϵ expression (undetectable by western blot; n=12). P-GP expression was determined cytometrically using MRK16 antibody staining (40).

To determine whether DNR resistance in the PKCϵ overexpression cell lines could be explained by P-GP-mediated drug efflux, we first evaluated P-GP expression by flow cytometry (**Figures 5B, C**). PKCϵ overexpression led to a >3.0-fold increase in P-GP expression compared to the control cell lines (**Figure 5D**), suggesting that PKCϵ-mediated DNR resistance is associated with P-GP upregulation. To establish a causal relationship between PKCϵ and P-GP, inhibition assays were conducted using the selective P-GP inhibitor Zosuquidar hydrochloride [ZSQ (48)]. In the PKCϵ overexpression cell lines, ZSQ increased DNR accumulation to levels equivalent to the control cell lines (**Figure 6A**), whilst there was no significant effect on DNR accumulation in the control lines. Furthermore, ZSQ treatment overcame PKCϵ-mediated resistance, restoring DNR sensitivity to that of the control lines (**Figure 6B**). Importantly, ZSQ treatment alone did not significantly impact the growth, viability, or autofluorescence of any of the cell lines investigated (**Supplementary Figures 13B, C**). Together these data demonstrate that P-GP drug efflux fully accounts for the PKCϵ-mediated DNR resistance observed.

Overexpression systems tend to give rise to much higher levels of protein expression than is observed in physiological or diseased states. Therefore, the relationship between high PKCϵ expression and P-GP expression was also assessed in the TCGA 2013 dataset of AML patient samples. No significant relationship between *P-GP* and *PKCϵ* mRNA was observed (**Supplementary Figure 14**), suggesting that a transcriptional mechanism of P-GP regulation by PKCϵ may be unlikely to fully account for our observations. A poor correlation between P-GP mRNA and protein expression has previously been reported in myeloid leukemia cells (49) and interrogation of the DEPMAP portal for all 44 AML cell lines also showed no positive correlation (Pearson = -0.303). This prompted us to examine the relationship between PKCϵ and P-GP protein expression in AML14/15 patient samples. Here we found the proportion of patients with above median P-GP expression was 2.8-fold higher in patients overexpressing PKCϵ than in those with low PKCϵ protein levels (**Figure 6C**). Although a larger patient cohort is required to determine the statistical significance of this observation, this does support the cell line analysis in indicating a post-translational link between PKCϵ overexpression and P-GP expression in AML.

P-GP activity has also been reported to be post-translationally regulated. P-GP contains several PKC consensus phosphorylation sites and despite conflicting evidence, phosphorylation of P-GP by PKC isoforms has been associated with P-GP activation and drug efflux (21, 23). To determine whether PKCϵ overexpression might confer DNR resistance through promoting P-GP activity, the impact of PMA, a potent PKC agonist, was assessed. PMA treatment of KG-1 cells (which endogenously express P-GP) significantly reduced DNR accumulation compared to DNR treatment alone (**Supplementary Figure 15A**). Furthermore, this reduction was antagonized by the P-GP inhibitor, ZSQ (**Supplementary Figure 15A**). In contrast, PMA treatment showed no impact on DNR accumulation in the control or PKCϵ overexpression cell lines (**Supplementary Figures 15B–E**) suggesting that, in these lines, activation of endogenous levels of P-GP are insufficient to account for increased pump activity which is more likely attributable to the increased expression of P-GP in these cells (**Figures 5B-D**), though the fact that PMA did not promote pump activity in the overexpressing lines suggests that PKCϵ is also optimally stimulating P-GP in these cells.

## Discussion

Despite evidence that PKC signaling plays a role in hematopoietic cell differentiation and survival (8, 50–54), little is known about the role of PKCϵ in hematological malignancies. To address this, we determined PKCϵ expression in two independent AML patient cohorts and showed that PKCϵ is frequently overexpressed. PKCϵ was associated with poor OS and DFS (mRNA) and CR-induction (protein), suggesting that PKCϵ could be an indicator of poor treatment response; findings which are supported by observations in solid cancer (4) and a study describing a risk prediction model which incorporated multiple AML molecular datasets (RNAseq, methylation, GEP and SNP) (55). We have no data that indicates what is controlling the regulation of *PRKCE* transcription in AML. While we did identify a study in breast cancer cell lines that highlighted a role for STAT-1 and Sip-1 in regulating PKCϵ transcription (56), we found no correlation with the expression of either of these factors with *PRKCE* expression in AML (data not shown).

Having determined that PKCϵ is frequently upregulated in AML, functional connotations were investigated. Analysis of PKCϵ mRNA and protein in hematopoietic progenitor cells showed an expression profile indicative of a role in myeloid cell differentiation. Functionally, knockdown of PKCϵ in HSPC had no significant impact apart from growth inhibition of granulocyte progenitors which suggests that its role may be redundant in this context. Conversely, PKCϵ overexpression in human HSPC impaired growth and promoted monocyte differentiation. This latter phenotype is concordant with the literature regarding other PKC isoforms (51, 52, 57) but is inconsistent with a role for PKCϵ in leukaemogenesis which is characterized by arrested myeloid development.

We next modelled the impact of PKC overexpression in the context of AML. PKCϵ overexpression significantly reduced the growth of U937 and HEL cells, potentially because of perturbed cell cycle progression. Although PKCϵ overexpression has been associated with pro-proliferative phenotypes (3, 58), PKC isoforms have also been associated with G2-phase delay and ERK-dependent upregulation of p21; a negative regulator of cell cycle progression (59, 60). We also found that PKCϵ overexpression conferred selective resistance to DNR. Given that an antioxidant role for PKCϵ has previously been described (14, 61), we first investigated whether PKCϵ-mediated resistance could be explained by the capacity of DNR to promote oxidative stress (43). However, we were unable to demonstrate enhanced antioxidant capacity in cells overexpressing PKCϵ. Instead, further work showed PKCϵ-mediated DNR resistance was accompanied by reduced intracellular DNR accumulation. DNR is a substrate for several efflux pumps, including P-GP (62) which is associated with reduced CR induction in AML (45) and reduces intracellular DNR (46). Furthermore, associations between PKCϵ and P-GP expression have previously been implicated in prostate and breast cancer cells (63, 64). In the present study, both PKCϵ overexpression AML lines showed P-GP upregulation by flow cytometry. Functional P-GP inhibition, with ZSQ, restored DNR accumulation and ablated DNR resistance in the PKCϵ overexpression cell lines. This demonstrates that P-GP drug efflux fully explains PKCϵ-mediated DNR resistance.

We also attempted to determine the effect of PKCϵ knockdown on drug sensitivity in AML cell lines and in primary AML patient-derived lines, however none of the cells in these panels demonstrated overexpression of PKCϵ making the experiments difficult to interpret. Specifically, in our panel of 10 AML lines only 3 showed detectable PKCϵ expression (Mv-4;11, U937 and OCI-AML5; **Supplementary Figure 16**) and none demonstrated overexpression, unsurprisingly while we achieved knockdown in U937 and Mv-4;11 (**Supplementary Figure 17**) we observed no significant impact on cell growth, viability or chemosensitivity (**Supplementary Figures 18, 19**). A similar outcome was obtained for our panel of 8 primary AML patient-derived lines (**Supplementary Table 4**) in the 2 lines which had detectable (though again modest) expression of both PKCϵ and P-GP (**Supplementary Figure 20**) knockdown also had no significant effect on drug sensitivity (**Supplementary Figure 21**); additionally, as ZSQ also had no impact on DNR sensitivity, these data suggest that P-GP was inactive in these cells (with the caveat that there was insufficient material to validate the extent of knockdown in these cells). Given the prevalence of PKC overexpression *in vivo*, these findings suggest that overexpression of PKCϵ is not sustained or is incompatible with *in vitro* culture of AML cells making the consequences of knockdown difficult to model. We also investigated the potential role of PKCθ which was co-expressed with PKCϵ in AML cell lines; however combined knockdown of both isoforms in OCI-AML5 cells also failed to impact drug sensitivity (data not shown).

P-GP expression is regulated at an mRNA and protein level (49, 65). No evidence for transcriptional regulation of P-GP by PKCϵ was observed in AML patient samples from the TCGA 2013 dataset; however, at a protein level, high PKCϵ was associated with high P-GP expression in AML cell lines and patient samples, indicating a post-translational mechanism of regulation. Promoting endogenous PKC activity using the cPKC and nPKC agonist, PMA, suggested that activation of endogenous P-GP activity alone was insufficient to explain DNR resistance and that increased expression of P-GP mediated by PKCϵ represents the main driver of this phenotype though we cannot rule out that activation of P-GP may also be a contributing factor.

The fact that Ara-C is not transported by P-GP is consistent with the observed selective effect of PKCϵ overexpression on increasing resistance to DNR. In patients, these agents are administered in combination, with Ara-C being infused continuously and DNR administered in pulses, which raises the question of the overall effect of PKCϵ overexpression under these circumstances. These pharmacokinetic conditions are difficult to model *in vitro* where drug levels remain largely static, we are therefore currently attempting to model the impact of PKCϵ overexpression on such a treatment regimen *in vivo.*

Overall, this study demonstrates that PKCϵ upregulation occurs frequently in AML, is a poor prognostic indicator associated with reduced patient responses to induction therapy and could contribute to poor outcomes by decreasing leukemia cell DNR chemosensitivity through promoting P-GP expression and drug efflux. Whilst clinically applicable inhibitors have yet to be generated, these data suggest that selective inhibition of PKCϵ may be effective in improving drug responsiveness in AML patients.

## Authors Contributions

RN designed and carried out experiments, analyzed all data and co-wrote the manuscript. AM, AA, and AL helped to collect and process human cord blood. SD provided training and technical support. CS provided the P-GP protein expression data in AML14/15 patient samples as well as support with the analysis. AG provided patient data and materials. SK provided clinical related advice and edited the manuscript. RD and AT secured funding, contributed to experimental design, data analysis and co-wrote the manuscript. All authors contributed to the article and approved the submitted version.

## Data Availability Statement

The original contributions presented in the study are included in the article/**Supplementary Material**. Further inquiries can be directed to the corresponding author.

## Ethics Statement

The studies involving human participants were reviewed and approved by South East Wales Local Research Ethics Committee. The patients/participants provided their written informed consent to participate in this study.

## Funding

Funding for this study was provided by the School of Medicine, Cardiff University. AA and AL were also funded by the School of Medicine, Cardiff University. AM was funded by Cancer Research Wales, U.K. RD and AT were supported by Blood Cancer UK (15018). SK is funded by CRUK through the Cardiff Experimental and Cancer Medicine Centre (ECMC: C7838/A25173).

## Conflict of Interest

The authors declare that the research was conducted in the absence of any commercial or financial relationships that could be construed as a potential conflict of interest.

## Publisher’s Note

All claims expressed in this article are solely those of the authors and do not necessarily represent those of their affiliated organizations, or those of the publisher, the editors and the reviewers. Any product that may be evaluated in this article, or claim that may be made by its manufacturer, is not guaranteed or endorsed by the publisher.
