# Supplementary material for: Protein Kinase C Epsilon Overexpression Is Associated With Poor Patient Outcomes in AML and Promotes Daunorubicin Resistance Through p-Glycoprotein-Mediated Drug Efflux
Source: Front Oncol. 2022 May 30;12:840046. doi: 10.3389/fonc.2022.840046 (PMC9191576; doi:10.3389/fonc.2022.840046)
Supplement: Supplementary file 1 [file DataSheet_1.docx]

# Supplemental Methods

## Analysing *PKCε* mRNA expression in normal haematopoietic cells and AML patient samples

*PKCε (PRKCE)* mRNA expression data from human (MicroArray; GSE42519;(1)) and murine (GSE14833; (2) and GSE6506 (3, 4)) haematopoietic stem and progenitor cells was obtained from Bloodspot (5). For AML patient samples, *PKCε* mRNA expression was assessed using the TCGA, NEJM 2013 dataset (6). which was accessed using cBioPortal (7, 8).

*PKCε* (*PRKCE*) mRNA expression was determined in AML14 and AML15 NCRI UK clinical trials by Affymetrix DNA microarray The raw data from the Hu133A GeneChip^®^ was normalised using MAS5 or Robust Multi Array (RMA) analysis. . Two probe-sets were used (236459_at and 226101_at) as these had the highest correlation and the highest association with MAS5 present calls (20/22).

## Lentiviral Expression Constructs

The PKCε overexpression lentiviral construct haboured the *PRKCE* gene under the control of the *human eukaryotic translation elongation factor 1 α1* (*EF1A*) promoter was designed using VectorBuilder Inc, Chicago, IL, USA. This was upstream of a dual *enhanced green fluorescent protein* (*EGFP*) and *puromycin* resistance (*puro*^R^) genes linked by a T2A sequence, which were included as selectable markers, and placed under the control of a *human cytomegalovirus* (*CMV*) promoter. For all experiments an EGFP;T2A;Puro^R^ construct with and empty open reading frame was used as a control. Details of shRNA vectors are described below.

Supplementary Methods Table 1. Summary of the shRNA constructs used

Table outlining the shRNA constructs used in cell line and primary cell experiments. PKCε shRNA sequences were identified using the Broad Institute Genetic Perturbation Platform. Constructs were selected based on their adjusted score; an indicator of the knockdown efficiency and gene specificity of each shRNA construct, while a shRNA sequence with no mammalian targets was used as a control throughout. Multiple shRNA candidate sequences, targeting a range of regions across the target mRNA sequence were selected and subsequently inserted into pLKO.1 U6-based shRNA expression vector and purchased from Vector Builder Inc. *Abbreviations; 3UTR; 3-prime untranslated region, CDS; coding sequence; GFP; green fluorescent protein, N/A; not applicable.*

| **shRNA Target** | **Construct Number** | **Clone ID** | **Target Sequence (5’🡪 3’)** | **Match Region** | **Adjusted Score** | **Fluorescent Marker** | **Antibiotic resistance** |
| --- | --- | --- | --- | --- | --- | --- | --- |
| **Control** | N/A | N/A | CCGGCAACAAGATGAAGAGCACCAACTCGAGTTGGTGCTCTTCATCTTGTTGTTTTT | N/A | -N/A | GFP | Puromycin |
| **PKCε** | 485 | TRNC0000000845 | CCCTTCAAACCACGCATTAAA | CDS | 21.0 | GFP | Puromycin |
|  | 486 | TRNC0000000846 | CCACAAGTTCGGTATCCACAA | 3UTR, CDS | 5.67 | GFP | Puromycin |

## Western Blot Densitometric Quantification

Band intensity on documented membranes were measured by densitometric analysis using ImageJ (Fiji; v. 2.0.0.71), unless otherwise stated. To do this, a region of interest (ROI) was constructed around a specific band. From this, a histogram of peak intensity was generated, and a baseline of background intensity was set from the area surrounding the band within the ROI. The area under the curve was then calculated to give an arbitrary intensity value. The band intensities of the protein of interest were normalised to the band intensity of the loading control for each sample normalised to loading (GAPDH expression) and converted into fg/1000 cells by comparing the relative band intensity to a recombinant PKCε standard of a known concentration.

## Drug Sensitivity Reagents

Master stocks of each agent were generated according to the manufacturer’s instructions and are outlined in Table 1 below. Once generated, all stocks were stored at ‑20^o^C in 100 µL aliquots for long-term storage unless otherwise stated.

Supplementary Methods Table 2. Agents used in drug sensitivity assays

Table outlining the agents, concentrations and vehicle controls used in drug sensitivity assays. *Abbreviations: Ara-C; cytarabine, ATM; antimycin A, ATO; arsenic trioxide, DNR; daunorubicin, GOx; glucose oxidase, PMA; Phorbol 12-myristate 13-acetate; ZSQ; zosuquidar hydrochloride.*^1^ Concentrations refer to the diluent, when used as a vehicle control these were diluted at 1µL/100µL of culture.

| **Drug** | **Manufacturer** | **Preparation of Master and working stocks** | **Cat no.** | **Diluent/ vehicle control^1^** | **Concentration** |
| --- | --- | --- | --- | --- | --- |
| Ara-C | Sigma-Aldrich | A 119mM Ara-C stock was generated by dissolving 100 mg solid Ara-C in 3 mL PBS. From this 840 µL was diluted in 9.2 mL PBS to generate a 10 mM working solution. | C1768 | PBS | 0-800nM |
| ATM | Sigma-Aldrich | A 20 Mm solution was generated by adding 2.3 mL ethanol to 25 mg ATM. From this a 2 mM working stock was generated by adding 100 μL ATM (20 mM stock) to 900 μL DMSO using a positive displacement pipette. | A8674 | DMSO | 0-20µM |
| ATO | Sigma-Aldrich | A 5 mM stock was generated by dissolving c20mg of solid ATO in 31 mL tissue-culture grade water (0.65 mg/mL). This was passed through a 0.22 µm filter and stored at RT. | 71287 | PBS | 0-4µM |
| DNR | Cayman Chemical | A 20mM master stock was generated by dissolving 5mg of solid DNR in 443µL DMSO. | 14159 | PBS | 0-100nM |
| GOx | Sigma-Aldrich | GOx (specific activity 192U/mg) was solubilised at 1mg/mL (equivalent to 192,000mU/mL) in PBS. When thawed for use, aliquots were used once. | 49180 | PBS+1% (*w/v*) BSA | 0-100mU |
| PMA | Cayman Chemical | A 100μM master stock was prepared in PBS and subsequently diluted 1/10 in PBS to generate a 10μM working stock. | 400145 | PBS | 1μM |
| ZSQ | Cayman Chemical | A 1mM stock was generated by dissolving 1.57mL DMSO. This 1mM stock solution was further diluted 1/100 in DMSO to make a 10µM working stock. | 21533 | PBS+2.5% (*v/v*) DMSO | 100nM |

## Cell Cycle Analysis

Transduced AML cell lines were seeded at 2x10^5^ cells/mL and expanded for 48 hours before being harvested to 1 mL flow tubes and washed with 1 mL PBS. The washed cells were subsequently resuspended in PBS and fixed for 30 min on ice by adding 700 µL absolute ethanol. After fixation, the tubes were stored at ‑20^o^C overnight. The next day, the cells were centrifuged at 270*xg* and washed with 1 mL PBS before being resuspended in 50 µL staining buffer (PBS+0.5% (*w/v*) BSA+ 0.02% (*w/v*) sodium azide). The cells were then stained with 25 µL staining solution containing 40 µg/mL propidium iodide, and 0.1 mg/mL RNase (Sigma‑Aldrich) diluted in PBS, for 30 min at 37^o^C. Samples were acquired within 20 min of this incubation using the Accuri™ C6 Plus flow cytometer. For analysis, debris and doublets were excluded, before the cell cycle status was resolved using the fluorescence intensity of propidium iodide (Sigma-Aldrich). Cell cycle analysis was performed using the Multicycle AV DNA analysis tool plug-in for FCS Express (DeNovo, Pasadena, USA).

## Colony Assays

Myeloid colony forming assays were performed by limiting dilution in U bottomed 96-plates (0.3 cells/well) using transduced (GFP^+^) HSPC enriched for cells highly expressing CD34 (CD34^+^) by FACS on day 3 of culture. Cells were cultured in Iscove's Modified Dulbecco's Medium (IMDM; Fisher Scientific, Loughborough, UK) supplemented with 5 ng/mL IL-3, , SCF, G-CSF and GM-CSF and incubated at 37°C with 5% CO_2_. Following 7 days of growth, individual colonies (> 50 cells) and clusters (> 5, < 50 cells) were scored.

## TOPRO-3 Analysis

Harvested cells were stained with a TOPRO-3 staining solution (RPMI supplemented with 100 mM HEPES and 50nM TOPRO-3 (T3605; Invitrogen, California, USA). For flow cytometric analysis, debris acquired during sample acquisition, defined as TOPRO-3 negative events with a FSC of <5x10^4^, were excluded and viability and viable cell counts were determined from within the viable cell population of TOPRO-3 negative events as shown below. Absolute counts/mL were determined from a 10 µL fixed volume acquisition.


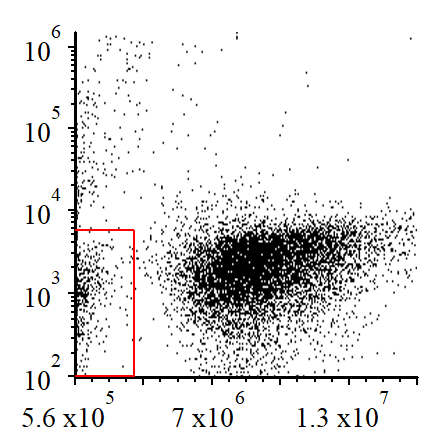

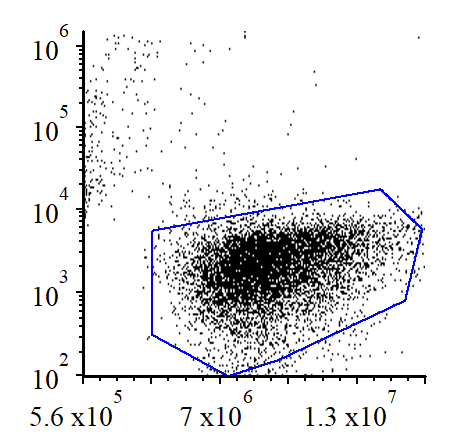


**Debris**

**FSC-A**

**FSC-A**

**TOPRO-3**

**A**

**B**

**TOPRO-3**

**Viable Cells**

Fig.1: Flow cytometric gating strategy used to evaluate the fold expansion and viability of AML cell lines

Representative bivariate plots demonstrating the gating strategy employed to determine the viability and fold expansion of AML cell lines. **(A)** Cells were stained with TOPRO-3 and debris, defined as TOPRO3 negative events with FSC-A properties of less than 5x10^4^, were excluded before the **(B)** viable counts and percentage viability were calculated from gating around TOPRO-3 negative cells.

## HSPC Immunophenotyping

The antibodies used to immunophenotype HSPC are outlined in Table . The gating strategy used for immunophenotypic analysis is outlined in Supplemental Figures (Fig. S4).

Supplementary Methods Table 3. Antibodies used for immunophenotypic analysis

Antibody supplier, clone, concentration, and manufacturer of antibodies used for immunophenotyping of AML cell lines and HSPC. *Abbreviations: APC; Allophycocyanin, PE; phycoerythrin.*

| **Antibody** | **Conjugate** | **Clone** | **Isotype** | **Concentration** | **Manufacturer** |
| --- | --- | --- | --- | --- | --- |
| IgG1 | PE | - | - | 5 ng/µL | BioLegend^®^ |
| CD11b | PE | ICRF4 | IgG1 | 5 ng/µL | BioLegend^®^ |
| CD13 | APC | WM15 | IgG | 5 ng/µL | BioLegend^®^ |
| CD14 | PE | HCD14 | IgG1 | 5 ng/µL | BioLegend^®^ |
| CD15 | PE | W6D3 | IgG1 | 5 ng/µL | BioLegend^®^ |
| CD34 | PE | 581 | IgG1 | 5 ng/µL | BioLegend^®^ |
| CD36 | Biotinylated | SMO | - | 1 ng/µL | Ancell Corporation |

**^1^** BioLegend®, California, USA, **^2^** Ancell Corporation, Minnesota, USA.

**References**

1. Rapin N, Bagger FO, Jendholm J, Mora-Jensen H, Krogh A, Kohlmann A, et al. Comparing cancer vs normal gene expression profiles identifies new disease entities and common transcriptional programs in AML patients. Blood. 2014;123(6):894-904.

2. Di Tullio A, Vu Manh TP, Schubert A, Castellano G, Månsson R, Graf T. CCAAT/enhancer binding protein alpha (C/EBP(alpha))-induced transdifferentiation of pre-B cells into macrophages involves no overt retrodifferentiation. Proc Natl Acad Sci U S A. 2011;108(41):17016-21.

3. Chambers SM, Boles NC, Lin KY, Tierney MP, Bowman TV, Bradfute SB, et al. Hematopoietic fingerprints: an expression database of stem cells and their progeny. Cell Stem Cell. 2007;1(5):578-91.

4. Berg JS, Lin KK, Sonnet C, Boles NC, Weksberg DC, Nguyen H, et al. Imprinted genes that regulate early mammalian growth are coexpressed in somatic stem cells. PLoS One. 2011;6(10):e26410.

5. Bagger FO, Sasivarevic D, Sohi SH, Laursen LG, Pundhir S, Sonderby CK, et al. BloodSpot: a database of gene expression profiles and transcriptional programs for healthy and malignant haematopoiesis. Nucleic Acids Res. 2016;44(D1):D917-24.

6. Ley TJ, Miller C, Ding L, Raphael BJ, Mungall AJ, Robertson A, et al. Genomic and epigenomic landscapes of adult de novo acute myeloid leukemia. N Engl J Med. 2013;368(22):2059-74.

7. Cerami E, Gao J, Dogrusoz U, Gross BE, Sumer SO, Aksoy BA, et al. The cBio cancer genomics portal: an open platform for exploring multidimensional cancer genomics data. Cancer Discov. 2012;2(5):401-4.

8. Gao J, Aksoy BA, Dogrusoz U, Dresdner G, Gross B, Sumer SO, et al. Integrative analysis of complex cancer genomics and clinical profiles using the cBioPortal. Sci Signal. 2013;6(269):pl1.
