# Supplementary material for: Protein Kinase C Epsilon Overexpression Is Associated With Poor Patient Outcomes in AML and Promotes Daunorubicin Resistance Through p-Glycoprotein-Mediated Drug Efflux
Source: Front Oncol. 2022 May 30;12:840046. doi: 10.3389/fonc.2022.840046 (PMC9191576; doi:10.3389/fonc.2022.840046)
Supplement: Supplementary file 2 [file DataSheet_2.docx]

Supplementary Material


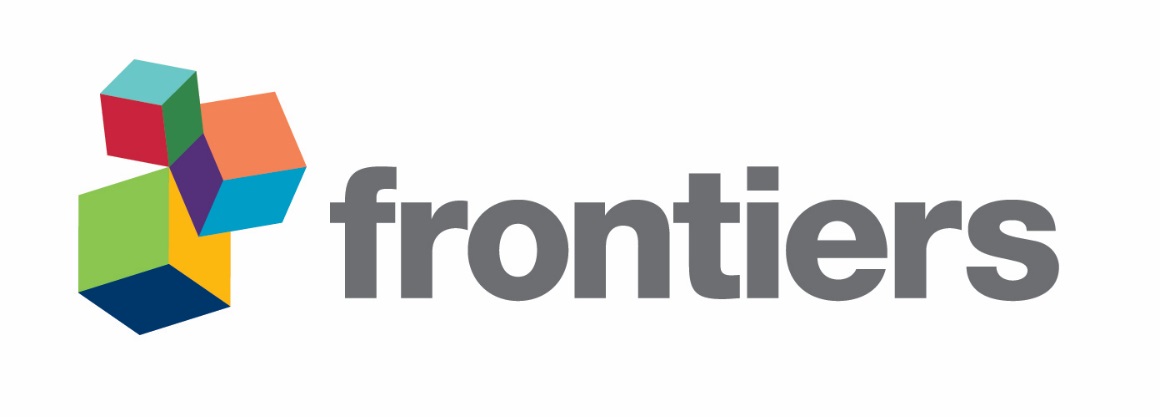


## Supplementary Figures

## Supplementary Figure 1. High *PKCε* expression in AML is not associated with FAB or specific molecular abnormalities


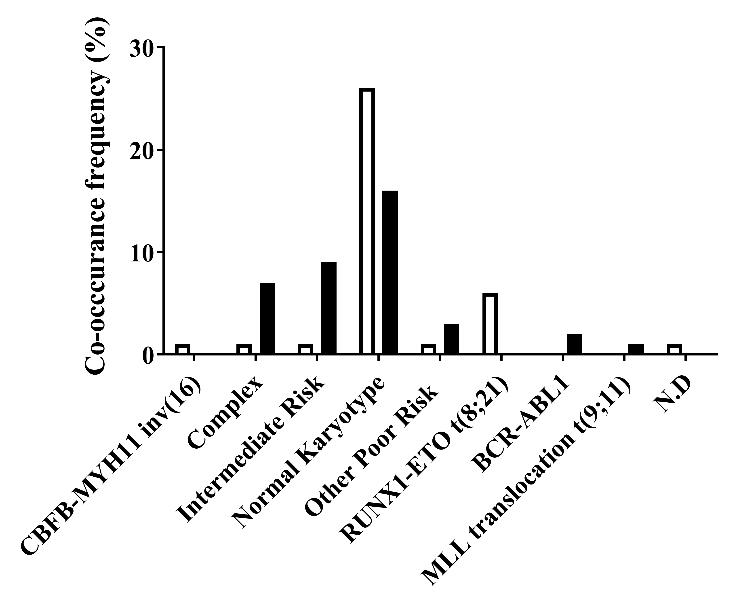

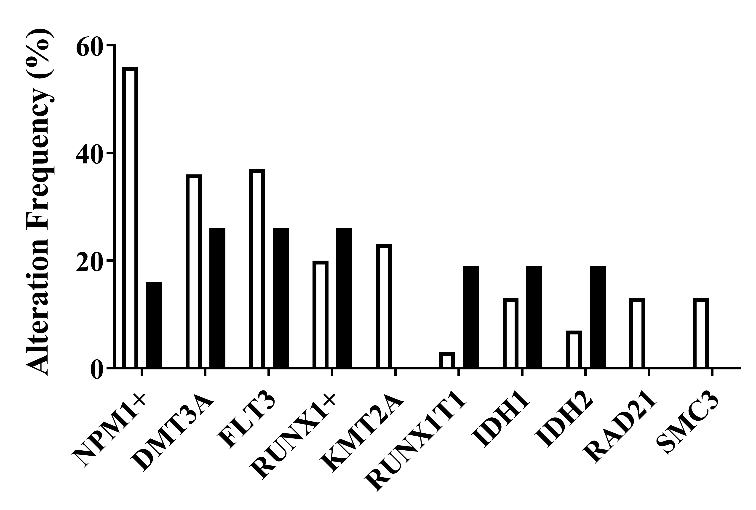


**Low *PKCε***

**High *PKCε***

**Low *PKCε***

**High *PKCε***

**B**

**A**

**C**

**Low *PKCε***

**High *PKCε***


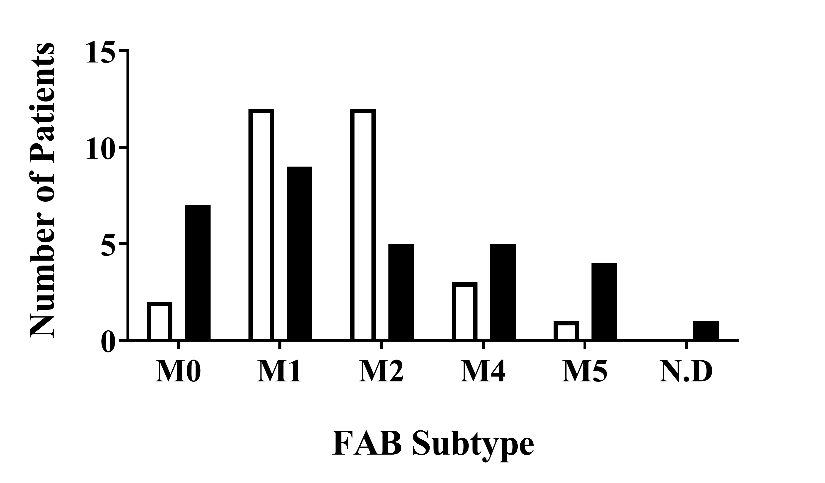


Bar chart representing the distribution of patients across the different **(A)** FAB subtypes of AML (M0‑M5) in the context of *PKCε* mRNA for patient samples from the TGCA 2013 dataset ((1)) with low (n=30) and high (n=31) *PKCε* mRNA expression, defined as the lower and upper quartiles, respectively. **(B)** Bar chart showing the cytogenetic and **(C)** molecular abnormalities with the highest co-occurrence frequency (%) in the context of *PKCε* mRNA expression for patient samples from the TGCA 2013 dataset ((1)) as described above.


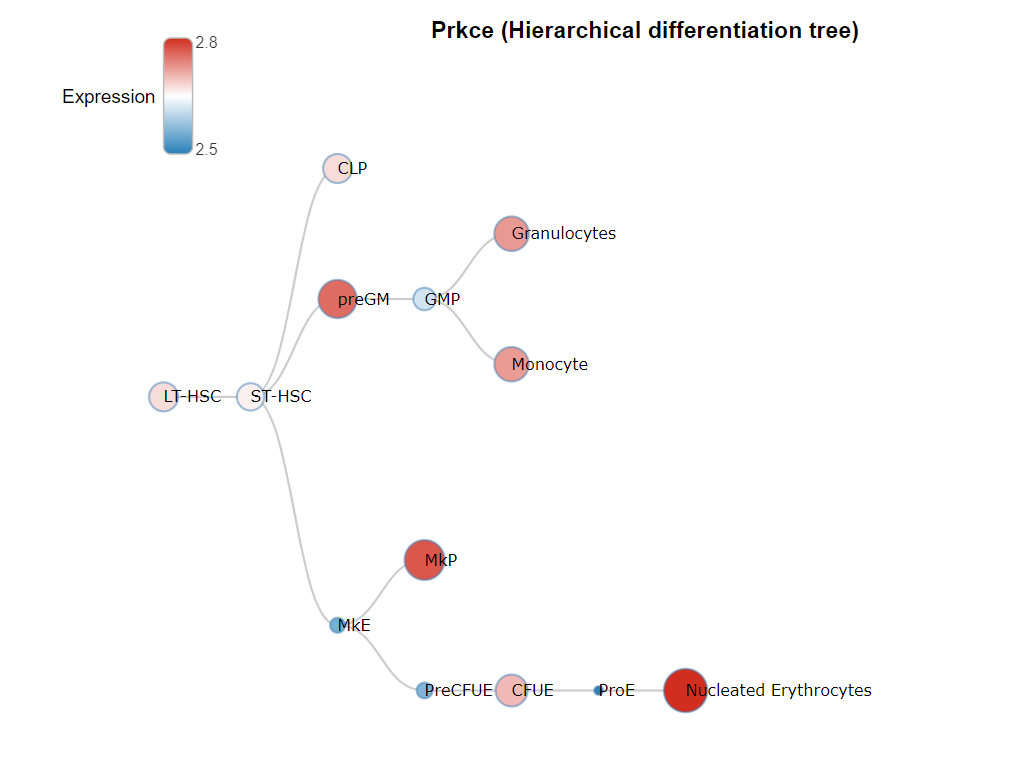

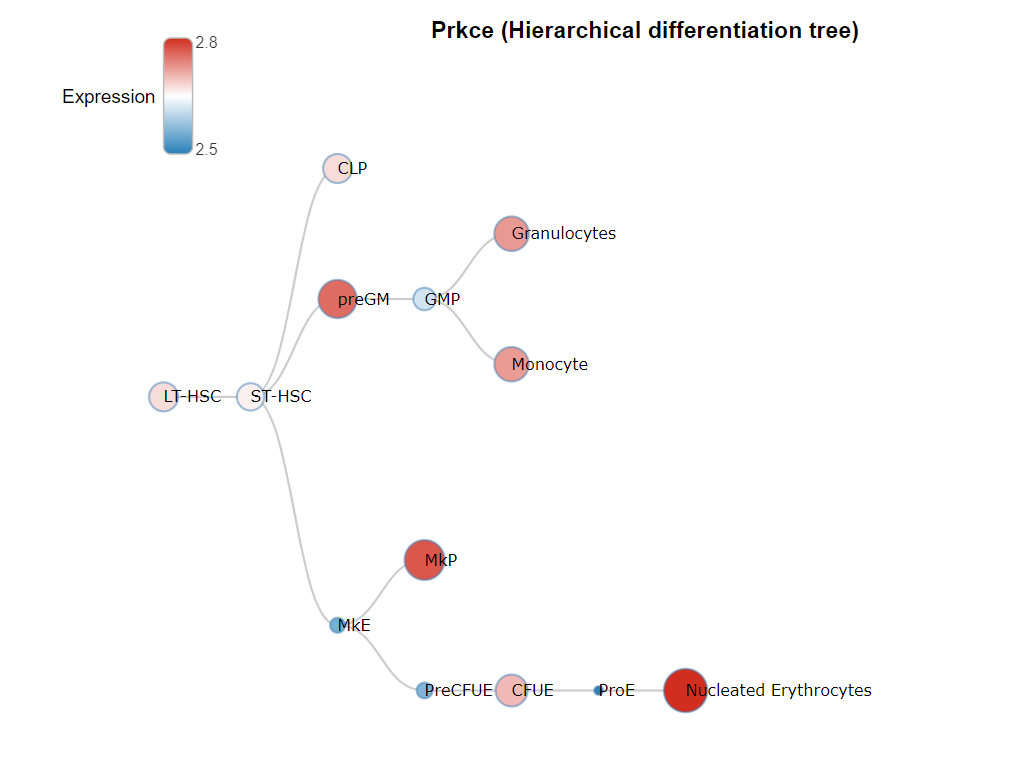


**A**

**B**


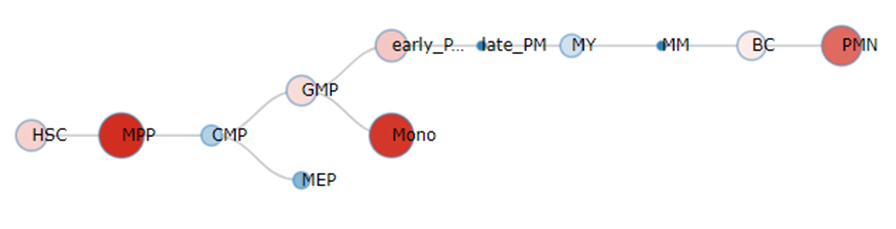

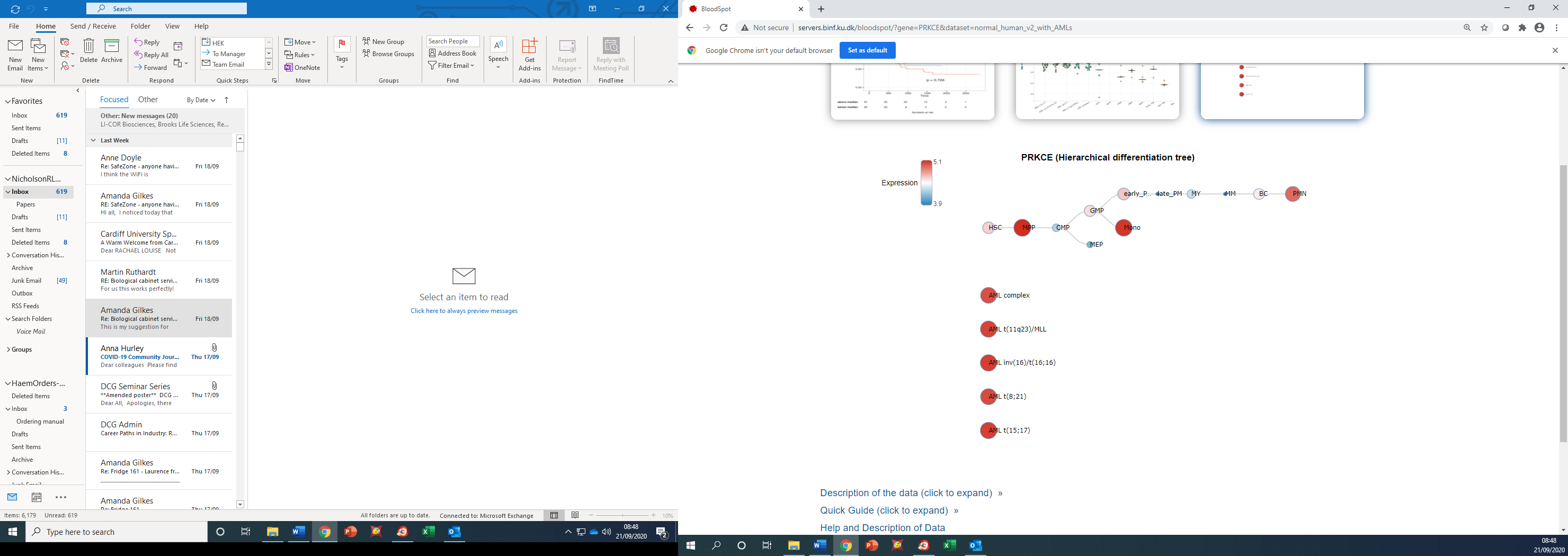


## Supplementary Figure 2. PKCε mRNA expression in haematopoietic progenitors

**(A)** Representative hierarchical differentiation trees showing *PKCε* mRNA expression (Log_2_ transformed) in human (probe-set 206248_at; GSE42519;(2)and (**B**) murine (probe-set 1437860_at; GSE14833; (3) and GSE6506 (4, 5)) haematopoietic progenitor subpopulations, determined by microarray using Affymetrix Human Genome U133 Plus 2.0 Array and Affymetrix Mouse Genome 430 2.0 Array respectively. Data and hierarchical tree were accessed using Bloodspot (6).

**Control**

**485**

**486**

**PKCε shRNA constructs**

**PKCε**

**GAPDH**

**A**

**B**


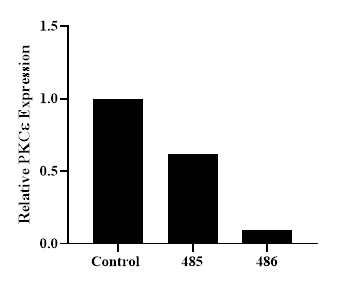


**PKCε shRNA constructs**


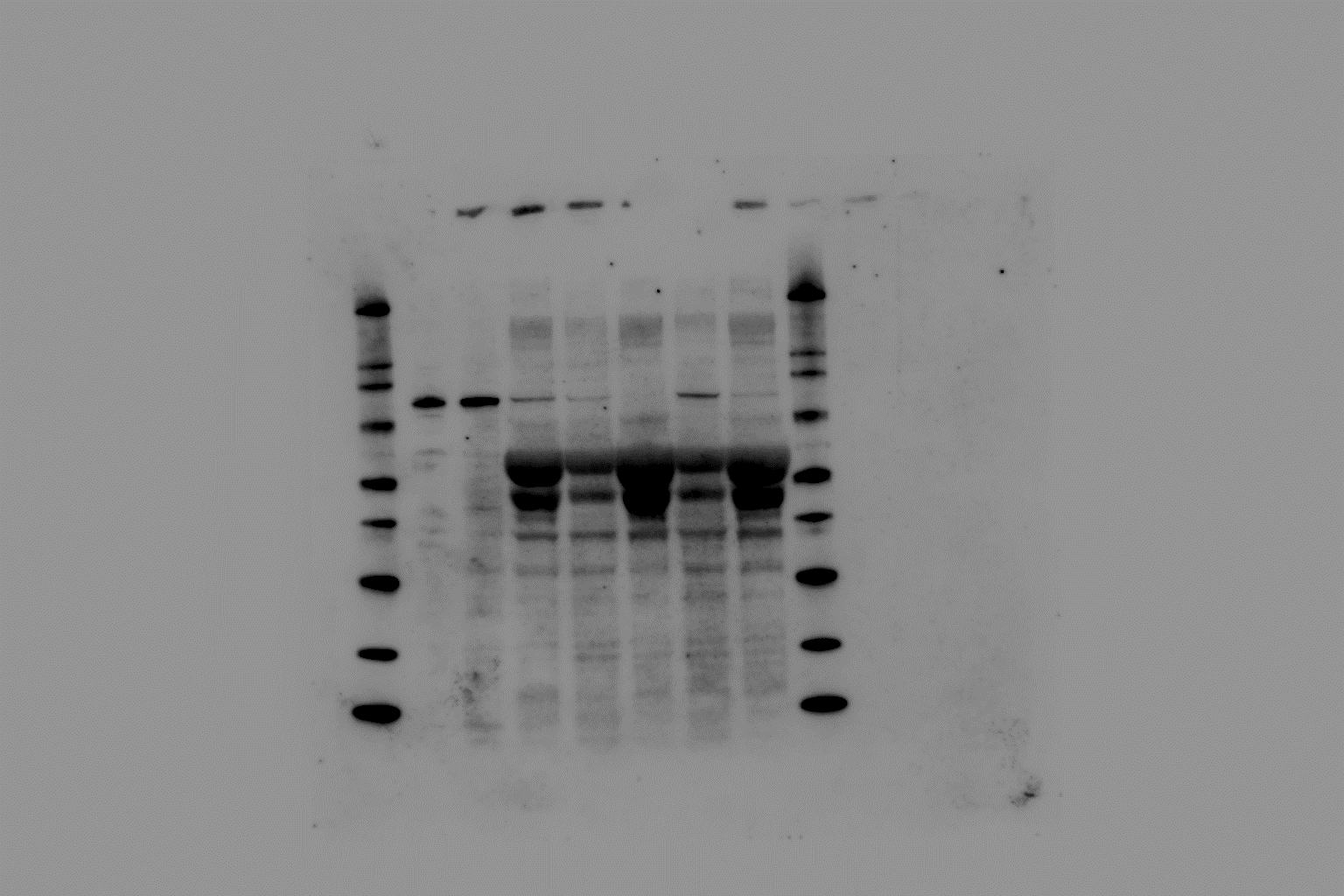

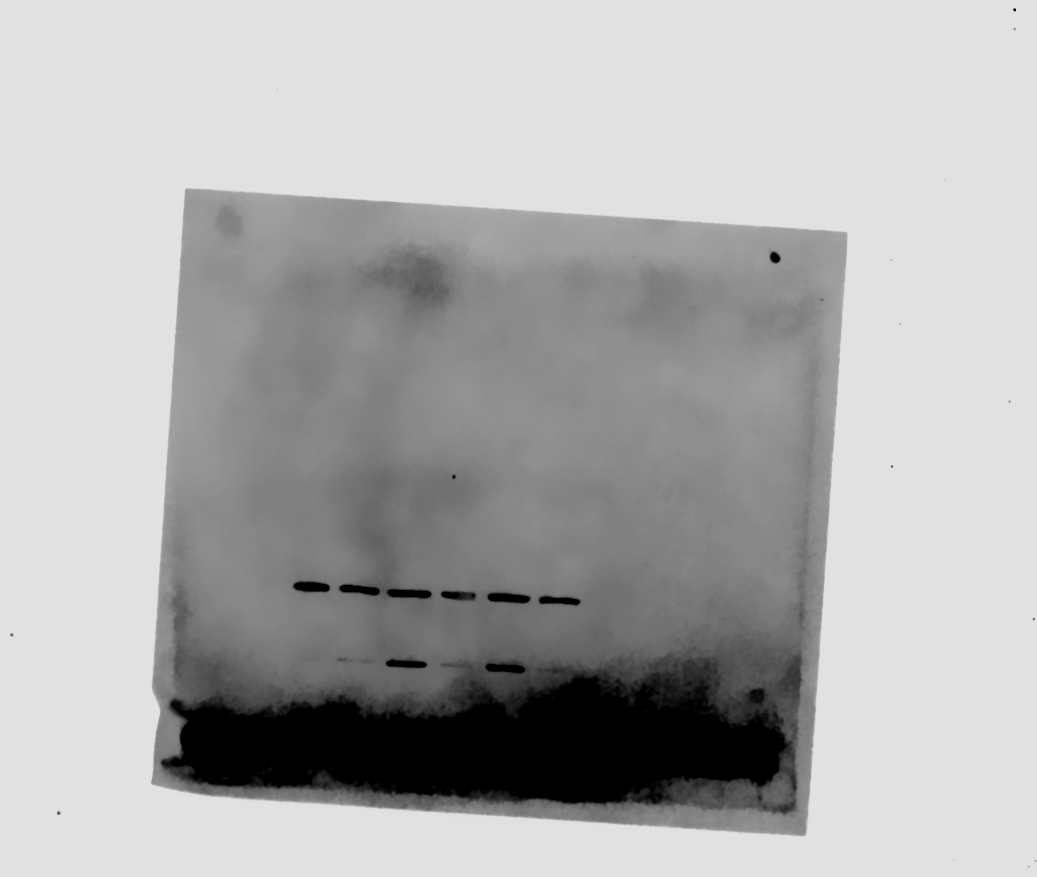


**Relative PKCε Expression**

## Supplementary Figure 3. Validating PKCε knockdown efficiency in HSPC

**(A)** Western blot and **(B)** densitometric quantification of PKCε (MW-84kDa) expression in HSPC transduced with the control or PKCε-targeted shRNA constructs (485 and 486) (described in Supplementary Methods) after 10 days of culture (see Materials and methods). Before lysate generation, the cells were enriched (>90%) by FACS based on GFP expression to remove any un-transduced cells. PKCε expression was detected using the Cell Signaling Technologies antibody (2B10; Supplementary Table 3) and is shown alongside GAPDH (MW-36kDa) expression, which was used as a loading control and was detected using the ThermoFisher Scientific antibody (GA1R; Supplementary Table 3). Densitometry analysis was conducted using Image J (Fiji) as described in the Materals and Methods. PKCε expression was calculated relative to loading (GAPDH expression) and normalised to the expression of HSPC cells transduced with the control shRNA construct; n=1.


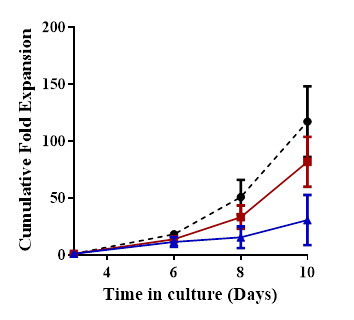


**A**

**B**

**C**

**Granulocytes**

**Monocytes**

**Total Culture**

**Control 485 486**

**Control 485 486**

**Control 485 486**

**

+


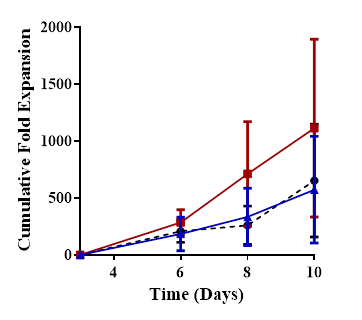

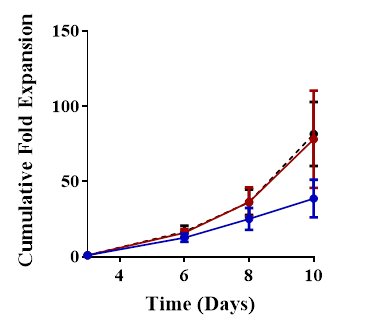

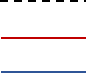

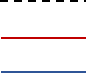

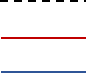


+++

## Supplementary Figure 4. PKCε knockdown reduces the fold expansion of granulocyte progenitors

*

Line graphs showing the cumulative fold expansion of HSPC **(A)** total culture, **(B)** monocyte (CD13^high^CD36^high^) and **(C)** granulocyte (CD13^low^CD36^low^) progenitors transduced with the control or PKCε-targeted shRNA constructs 485 and 486, as described in Supplementary Methods, over 10 days of culture (see Materials and methods). Analysis commenced at day 3 of culture; n=4; data represents the mean ± 1SD. Statistical significance between the control and knockdown cultures at each timepoint was determined using two-way ANOVA with Bonferroni post-test comparison and was deemed significant (*control vs 485; +control vs 486); *p<0.05; ** p<0.01, ***p<0.001.

## Supplementary Figure 5. PKCε knockdown does not significantly affect monocyte differentiation

**A**

**B**

**C**

**Control 485 486**

**Control 485 486**

**Control 485 486**


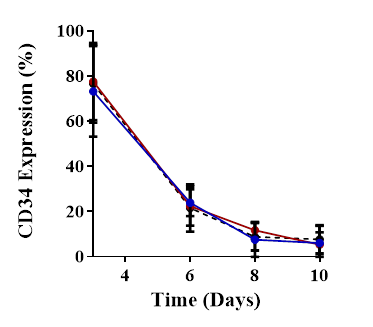

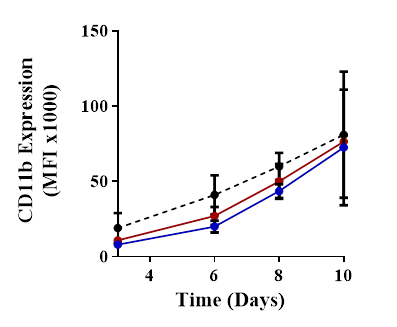

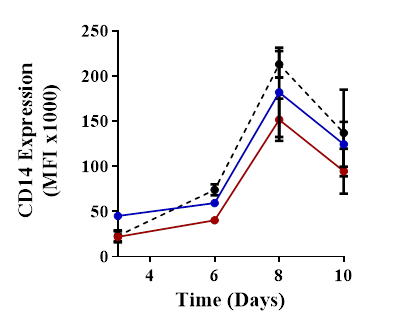

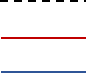

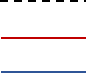

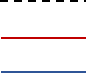


Line graphs showing the immunophenotypic profile of monocyte (CD13^high^CD36^high^) progenitor s transduced with the transduced with the control or PKCε-targeted shRNA constructs 485 and 486, as described in Supplementary Methods, over 10 days of culture (see Materials and methods). Analysis commenced at day 3 of culture; n=3, data represent mean ± 1SD. Cells were stained for **(A)** CD34, **(B)** CD11b and **(C)** CD14 expression; CD34 expression is represented by the proportion of positive cells (%) while CD11b and CD14 expression is represented using the fluorescence intensity (MFI x1000). Antibodies used for this analysis are outlined in Supplementary Table 3. Statistical significance was determined by two-way ANOVA with Bonferroni post-test comparison; and was deemed non-significant.

*

## Supplementary Figure 6. PKCε knockdown does not significantly affect granulocyte differentiation

**A**

**B**

**C**

**Control 485 486**

**Control 485 486**

**Control 485 486**


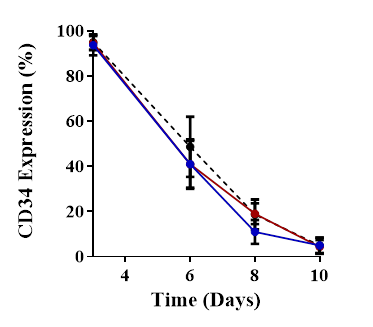

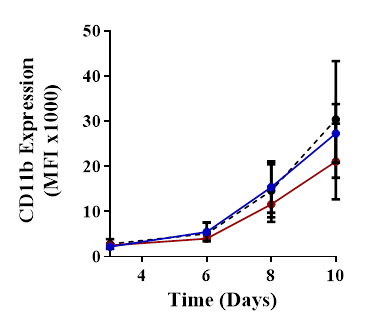

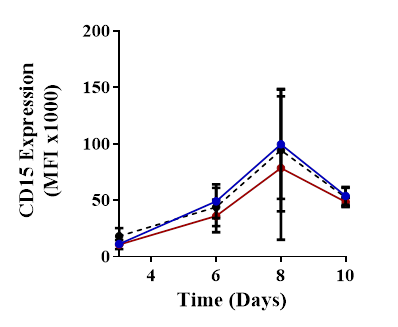

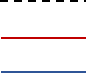

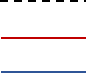

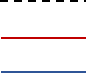


Line graphs showing the immunophenotypic profile of granulocyte (CD13^low^CD36^low^) progenitor cells transduced with the control or PKCε-targeted shRNA constructs 485 and 486, as described in Supplementary Methods, over 10 days of culture (see Materials and methods). Analysis commenced at day 3 of culture; n=3, data represent mean ± 1SD. Cells were stained for **(A)** CD34, **(B)** CD11b and **(C)** CD15 expression; CD34 expression is represented by the proportion of positive cells (%) while CD11b and CD15 expression is represented using the fluorescence intensity (MFI x1000). Antibodies used for this analysis are outlined in Supplementary Table 3. Statistical significance was determined by two-way ANOVA with Bonferroni post-test comparison and was deemed non-significant.

## Supplementary Figure 7. PKCε overexpression in HSPC


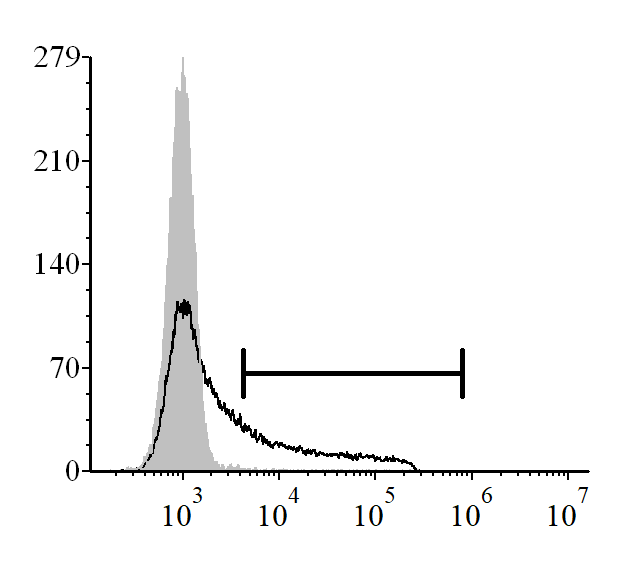

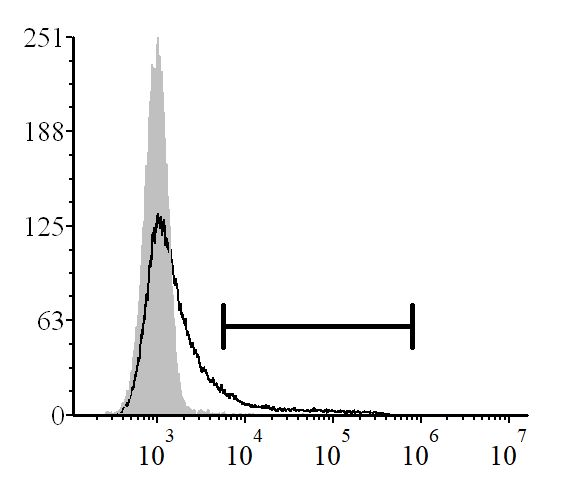


**Control**

**GFP**

**GFP**

**B**

**C**

**PKCε**

10%

Count

24%

Count

**HSPC**


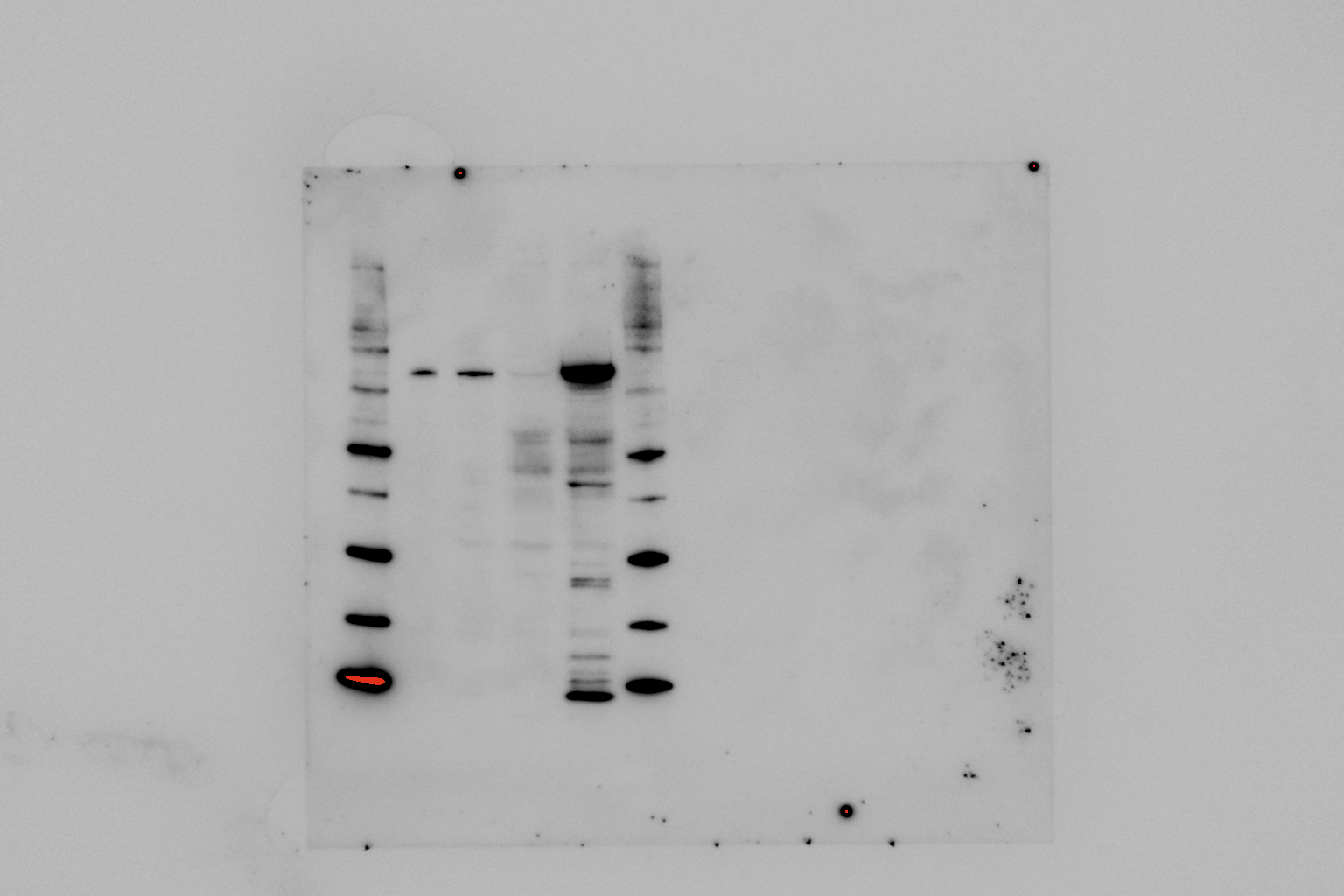


**Control**

**PKCε**

**Mv4;11**

**PKCε**

**GAPDH**


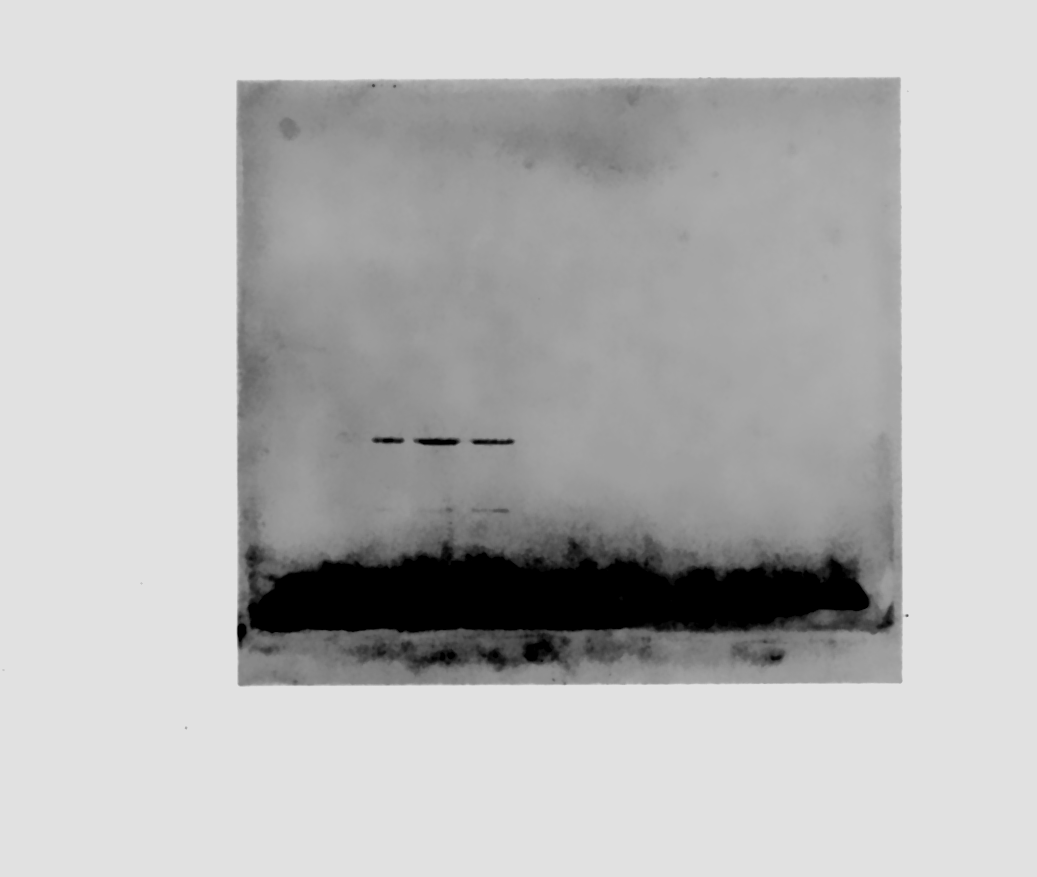


**A**


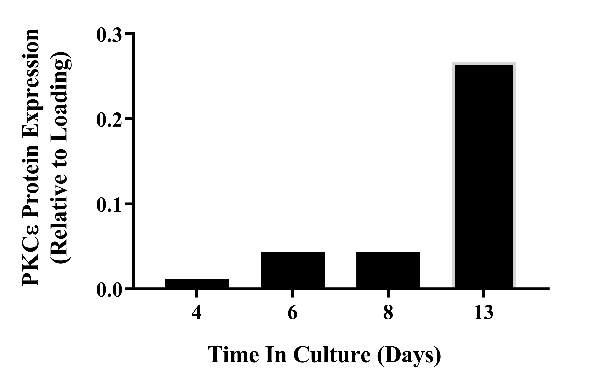


**(A)** Bar chart showing densitometry quantification of PKCε (MW-84kDa) expression in HSPC over 13 days of culture (corresponding western blot shown in Figure 2B). Densitometry analysis was performed as described in Material and methods and is display relative to loading (GAPDH expression). **(B)** Representative histograms showing the transduction efficiency of the control (top) and PKCε overexpression (bottom) constructs in HSPC following two days of lentiviral infection (day 3). GFP expression was used as a marker of successful transduction, where un-transduced HSPC were used to set the threshold for GFP expression (grey). **(C)** Western blot showing PKCε (MW-84kDa) expression in HSPC transduced with the control or PKCε overexpression constructs following 13 days of culture. Before lysate generation, the cells were enriched (>90%) by FACS based on their GFP expression. PKCε expression was detected using the Cell Signaling Technologies antibody (22B10; Supplementary Table 3). Mv4;11 cells were used as a positive control. PKCε expression is shown alongside GAPDH (MW-36kDa) expression, which was used as a loading control and detected using the ThermoFisher Scientific antibody (GA1R); n=1.

## Supplementary Figure 8. Gating strategy for analysing the impact of PKCε on myeloid development


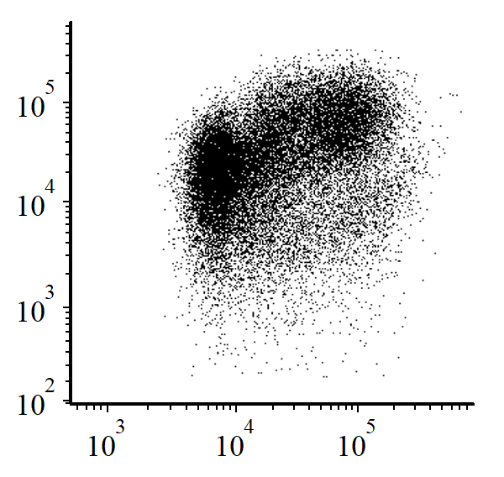

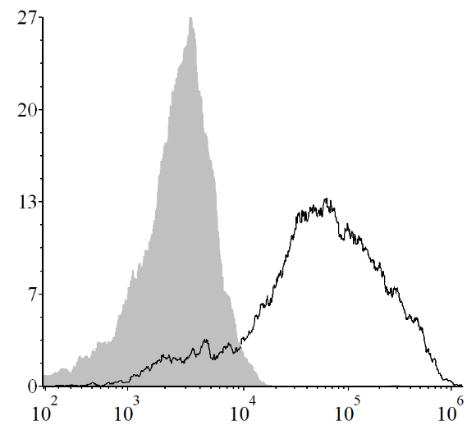


**Monocytes**

**Granulocytes**

**CD13**

**CD36**

**Erythrocytes**

**SSC-A**

**GFP**

**FSC-A**

**SSC-A**

**CD14**

**Counts4**

**A**

**B**

**C**

**D**

**IgG CD14**

**Non-Debris**

**GFP+**


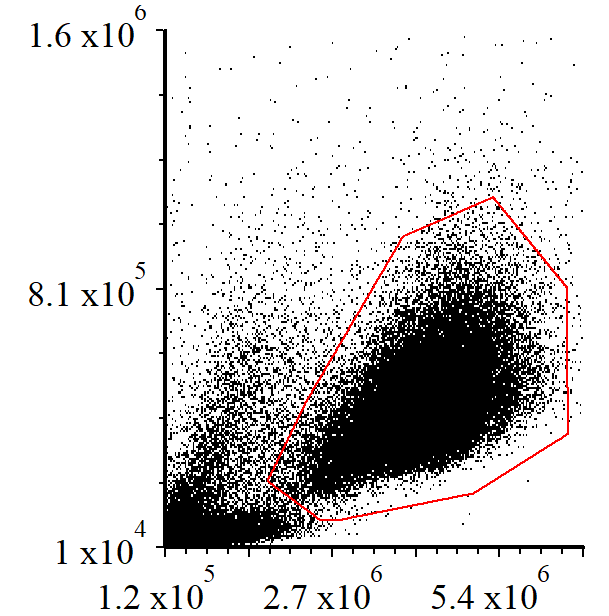

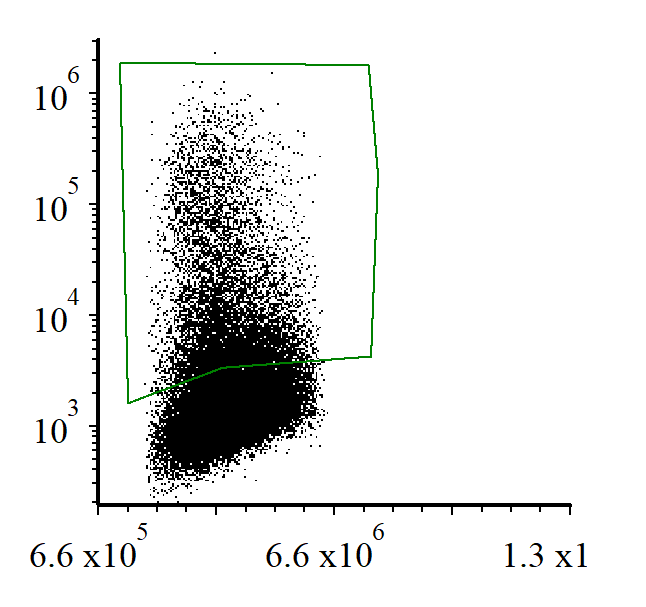


Representative bivariate plots and histogram outlining the gating strategy used for immunophenotyping HSPC. **(A)** Debris were excluded based on light scattering. **(B)** GFP expression was then used to limit subsequent analysis to transduced HSPC (GFP^+^). **(C)** Expression of the lineage discriminating markers CD13 and CD36 were used to resolve the monocytic, granulocytic and erythroid progenitor populations. **(D)** Within the monocytic and granulocytic progenitor populations the expression of maturation markers such as CD14 on monocyte progenitors were assessed using flow cytometric histograms using an appropriate isotype control (grey) to set background limit of fluorescence. Antibodies used for this analysis are outlined in Supplementary Table 3.


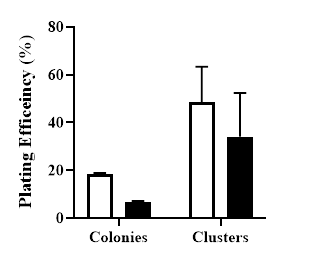


**Control PKCε**

## Supplementary Figure 9. PKCε overexpression reduces the colony formation capacity of HSPC

Bar chart showing the colony (>50 cells) and cluster (>5 to <50 cells) plating of transduced with the PKCε overexpression or control constructs following 7 days of culture. Following transduction, HSPC were enriched for early progenitors (GFP^+^CD34^+^CD38^‑^) by FACS before being plated in at U-bottomed 96 well plate (Supplementary Methods). Data represent mean±1SD (n=2).


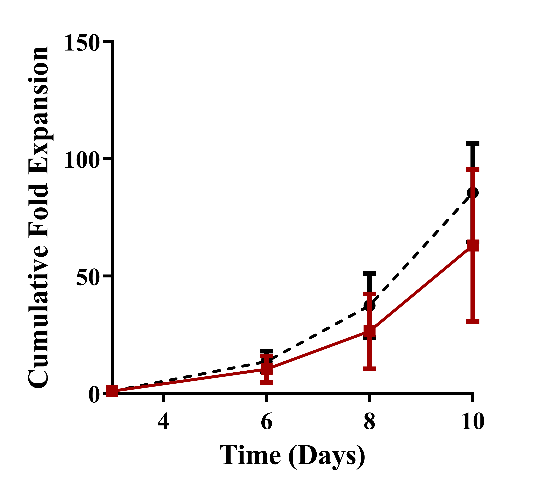


**C**


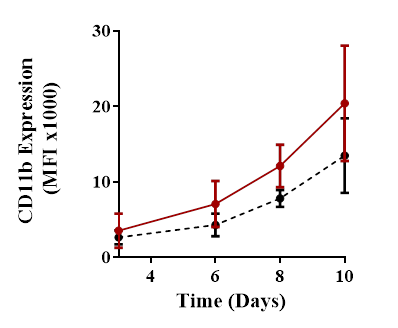

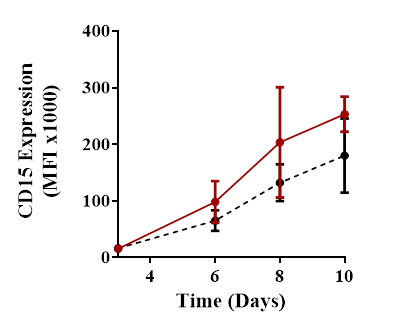


**A**

**B**

**Control PKCε**


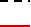


**Control PKCε**

**Control PKCε**


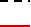

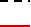


## Supplementary Figure 10. Effect of PKCε overexpression on granulocytic development

Line graph showing the **(A)** cumulative fold expansion and expression (MFI) of **(B)** CD11b and **(C)** CD15 on granulocytic progenitors (CD13^high^CD36^high^; Supplementary Figure 8) transduced with the control and PKCε overexpression constructs. Data represent mean±1SD (n>3).

**Control PKCε**

**

**

*

**A**

**B**

**U937**

**HEL**

**Control PKCε**


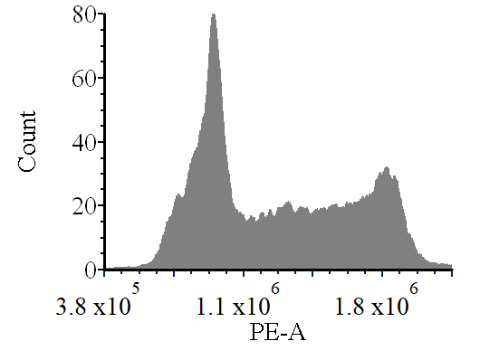

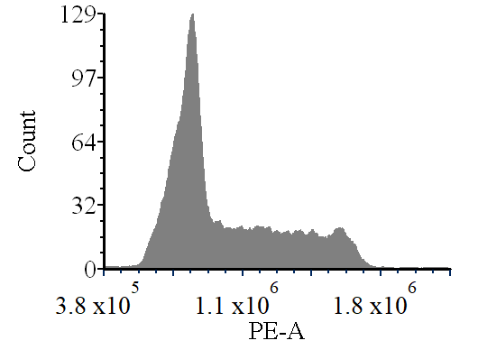

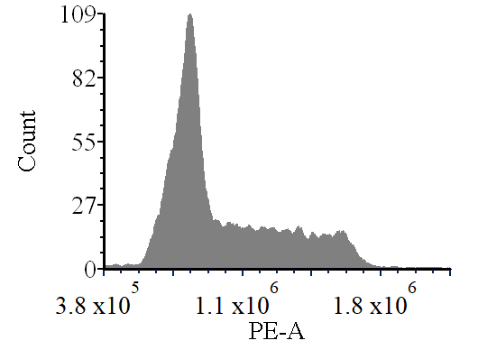

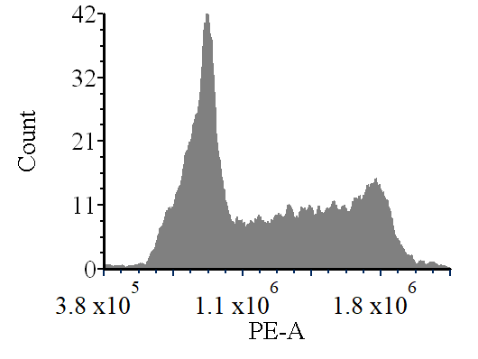


**Control**

**PKCε**

**Control**

**PKCε**

## Supplementary Figure 11. PKCε overexpression is associated with an increased proportion of cells in G2-phase of the cell cycle

Representative histogram (left) and bar charts (right) showing the proportion (%) of cells in the G1, S and G2 phases of the cell cycle, determined by propidium iodide (PI) staining (Supplementary Methods) for **(A)** U937 and **(B)** HEL cells transduced with the control or PKCε overexpression constructs. Bar chart data represents mean+1SD (n=3). Statistical significance was determined using a two-way ANOVA with Bonferroni post-test comparison, * p <0.05 , ** p<0.01.

## Supplementary Figure 12. PKCε overexpression does not confer resistance to ROS generating agents

**HEL**

**U937**

**A**

**B**

**Control**

**PKCε**

**Control**

**PKCε**

*

***

*


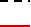

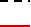


**C**

**Control**

**PKCε**

**Control**

**PKCε**


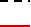

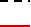


**Control**

**PKCε**

**Control**

**PKCε**


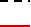

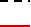


Line graphs showing the effect of increasing **(A)** glucose oxidase (GOx), **(B)** antimycin a (ATM) and **(C)** arsenic trioxide (ATO) concentration on the viability of U937 (left) and HEL (right) cells transduced with the control or PKCε overexpression constructs, following 48 hours of treatment. Viability was determined by flow cytometry using TOPRO-3 staining and normalised to cells treated with the vehicle controls; GOx and ATM n=3, ATO n=2; data represents mean±1SD. Statistical significance was determined using a two-way ANOVA with Bonferroni post-test comparison and was deemed significant;*p<0.05,***p<0.001.

## Supplementary Figure 13. DNR uptake and impact of ZSQ treatment on cell growth, viability and DNR sensitivity after 2 days of treatment


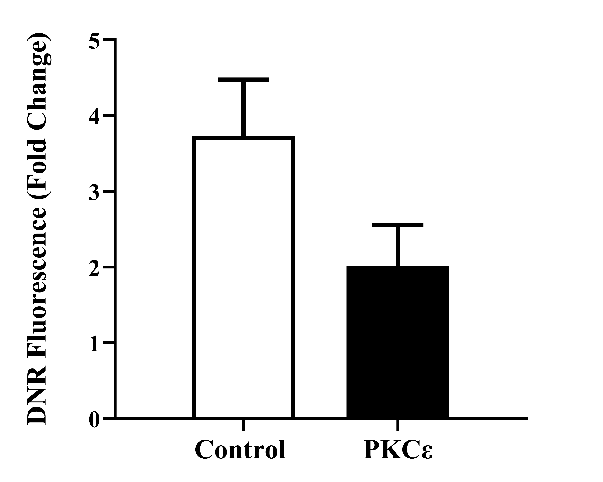

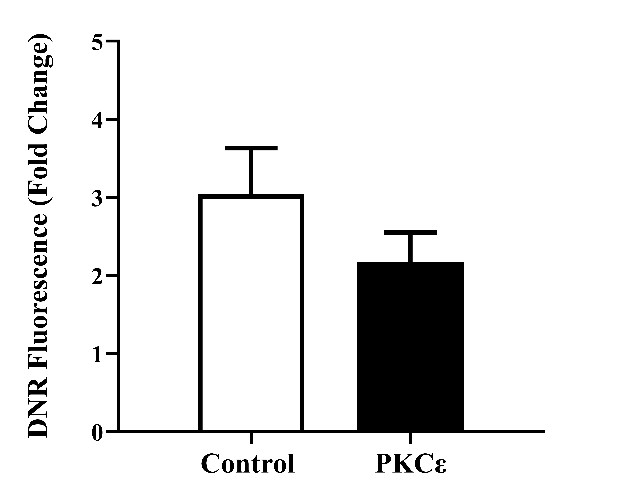


**HEL**

**U937**

*


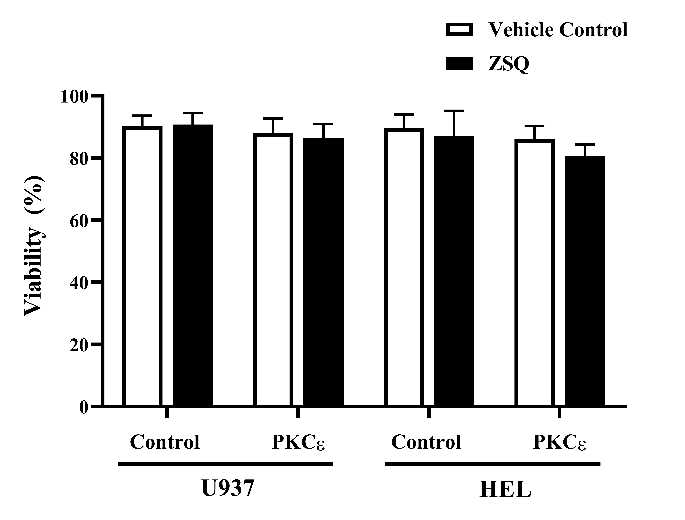

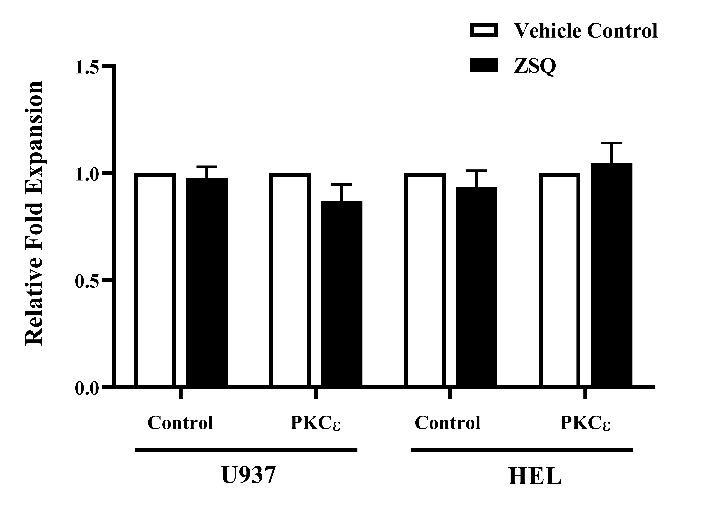


**A**

**B**

**C**

**D**


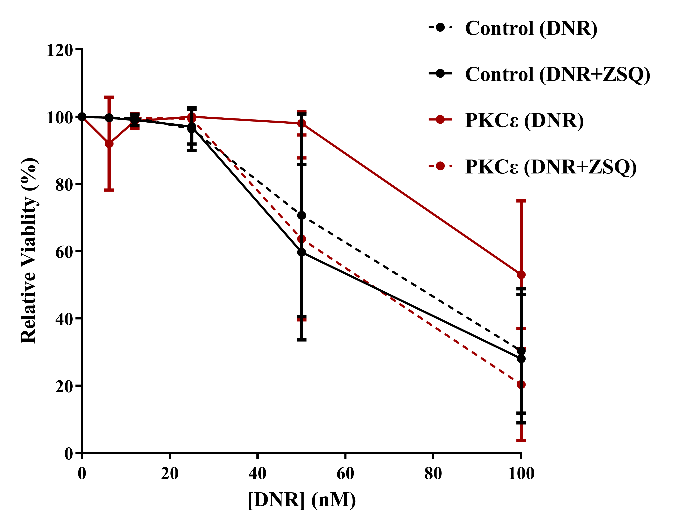

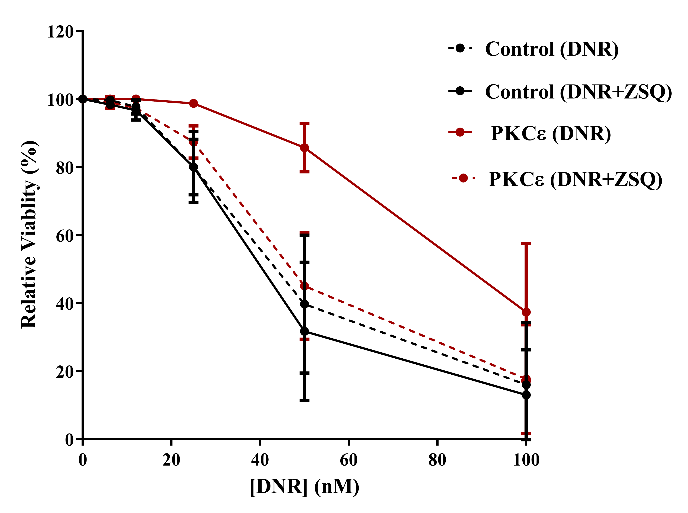


**HEL**

**U937**

**HEL**

**U937**

**(A)** Bar charts showing the DNR accumulation (fold change relative to cells treated with the vehicle control, (PBS)) of U937 (left) and HEL (right) cells transduced with the control and PKCε overexpression constructs (Supplementary Methods), following 2 days of treatment with 100 nM DNR. (**B**) Bar charts showing the effect of ZSQ treatment (100 nM) on the fold expansion and (**C**) the viability of U937 and HEL cells following 48 hours of treatment. Data represents mean+1SD (n=3). Statistical significance was determined using paired t-tests; *p<0.05. **(D)** Line graphs showing the dose response curves of U937 (left) and HEL (right) cells transduced with the control (black) and PKCε (red) overexpression constructs (Supplementary Methods), in response to 2 days of treatment with 0 nM -100 nM DNR alone (solid lines) or in combination with 100 nM ZSQ (dashed lines).


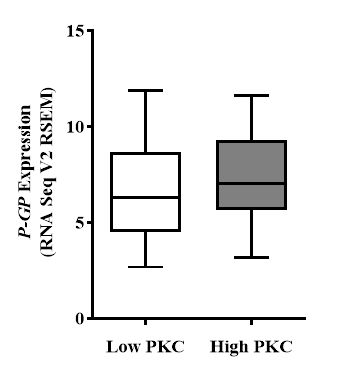


**Low *PKCε***

**High *PKCε***

## Supplementary Figure 14. *P-GP* mRNA expression is not associated with *PKCε* expression in AML cell lines and patient samples

Box and whisker plot showing *P-GP* mRNA expression (RNASeq V2 RSEM on a linear scale) in AML patients with low (lower quartile, n=37) and high (upper quartile, n=38) *PKCε* (*PRKCE*) expression from the TCGA 2013 dataset (1). The median is represented with the black line, the box represents the interquartile range, and the whiskers represent the minimum and maximum values.

## Supplementary Figure 15. PKC activity does not directly affect P-GP functionality

*

***

**KG-1**

**U937**

**A**

**B**

**C**

**D**

**E**

**HEL**

**Control**

**Control**

**PKCε**

**PKCε**


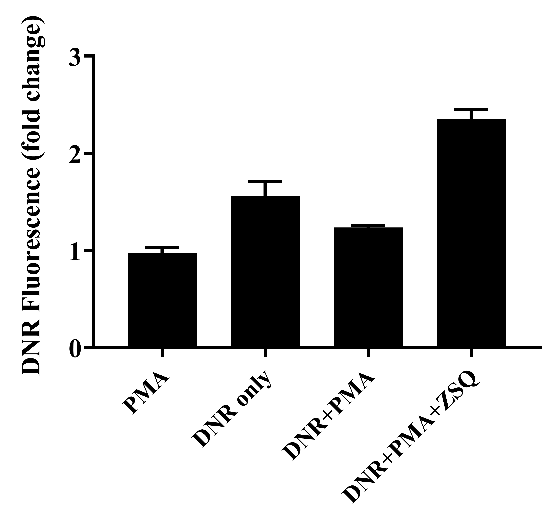

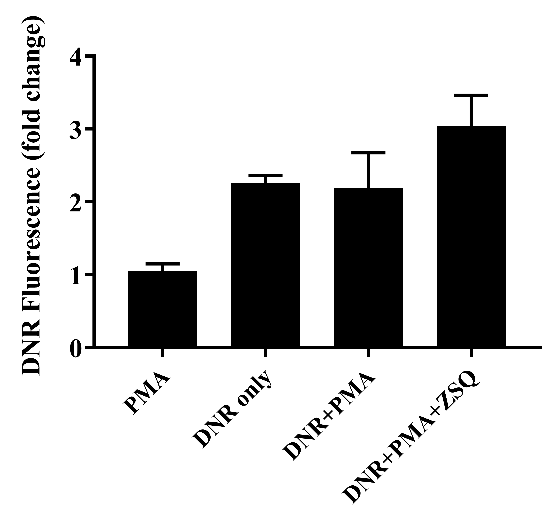

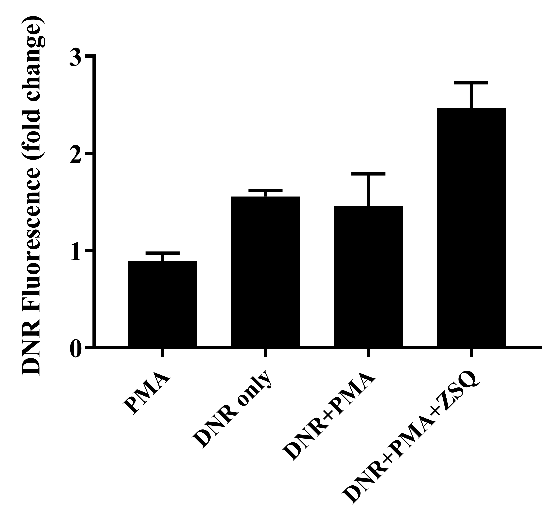

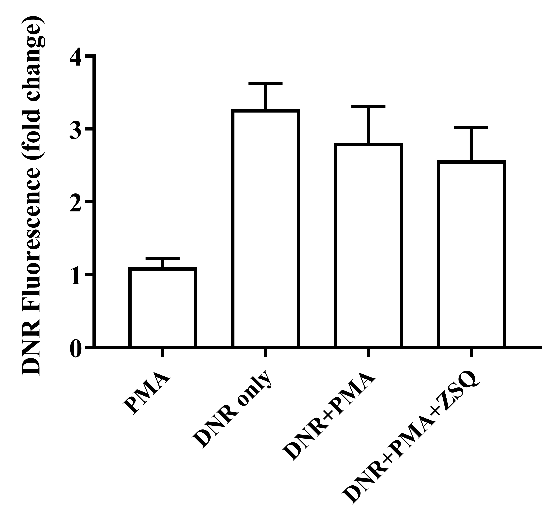

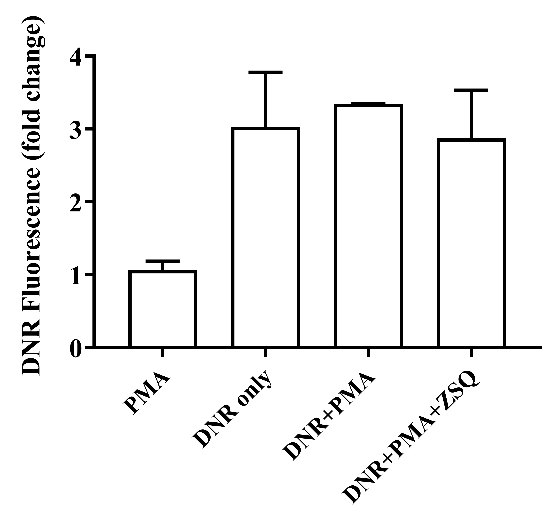


Bar chart showing the effect of PMA treatment (100nM) on DNR accumulation following 2 hours of treatment with 100nM DNR in **(A)** KG‑1 cells and **(B-E**) U937 and HEL cells transduced with the control (left) or PKCε overexpression (right) constructs. DNR accumulation was determined by measuring DNR fluorescence and was normalized to the fluorescence of cells treated with the vehicle control (PBS). Data represents mean+1SD; n=3. *p<0.05, ***p<0.001; statistical significance was determined using one-way ANOVA with Bonferroni post-test comparison, where comparisons were made to DNR treatment alone.


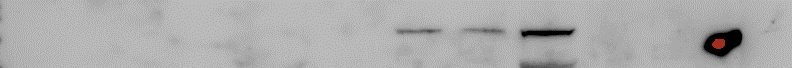

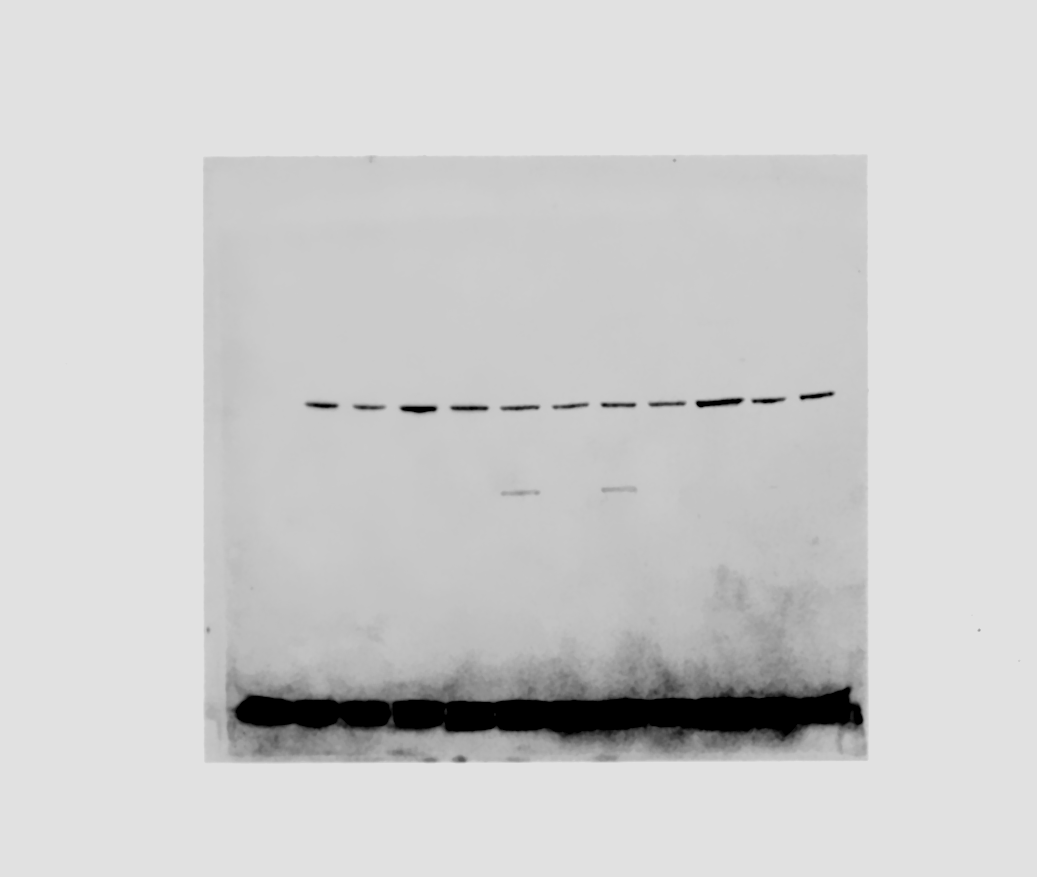


**NOMO-1**

**THP-1**

**HEL**

**HL-60**

**K562**

**Mv4;11**

**U937**

**OCIAML5**

**TF-1**

**SKNO-1**

**PKCε**

**GAPDH**

## Supplementary Figure 16. PKCε protein is heterogeneously expressed in leukaemia cell lines

Western blot image showing PKCε protein (MW-84kDa) expression in a panel of 10 leukaemia cell lines. PKCε expression was detected using the Cell Signaling Technologies antibody (22B10; Supplementary Table 3) and is shown alongside GAPDH (MW-36kDa) expression, which was used as a loading control and detected using the ThermoFisher Scientific GAPDH antibody (GA1R; Supplementary Table 3); n=1.

## Supplementary Figure 17. Validating PKCε shRNA knockdown efficiency in AML cell lines

**U937**

**Control**

**485**

**486**


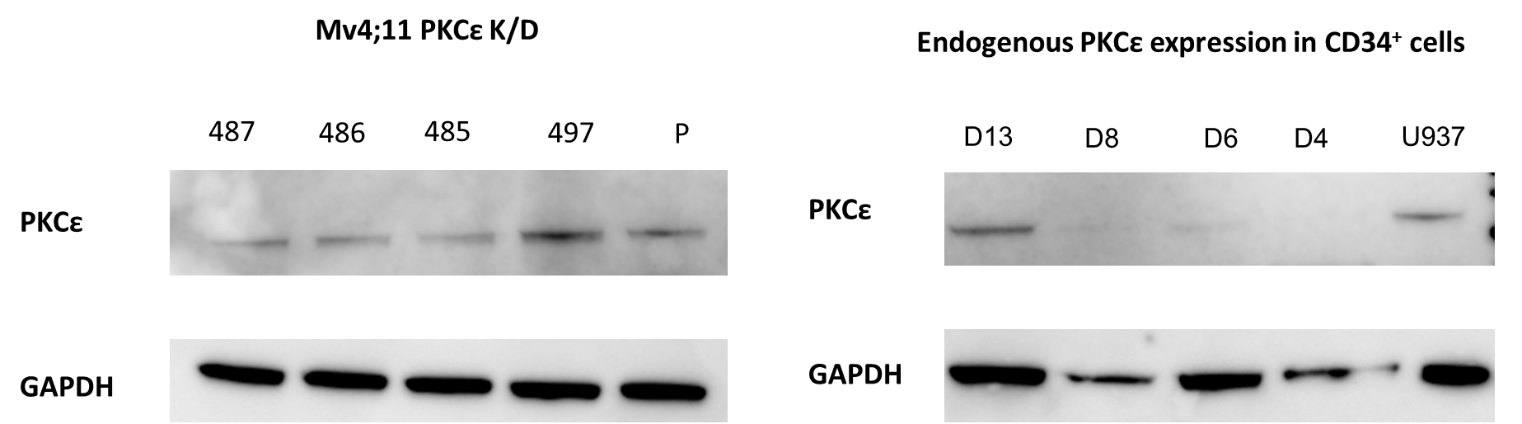

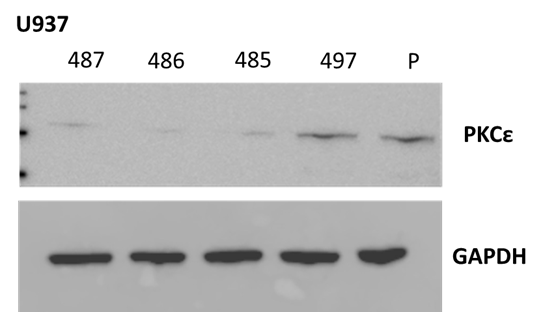


**Mv4;11**

**Control**

**485**

**486**

**PKCε**

**PKCε**

**GAPDH**

**GAPDH**

**A**

**B**

**PKCε shRNA**

**PKCε shRNA**


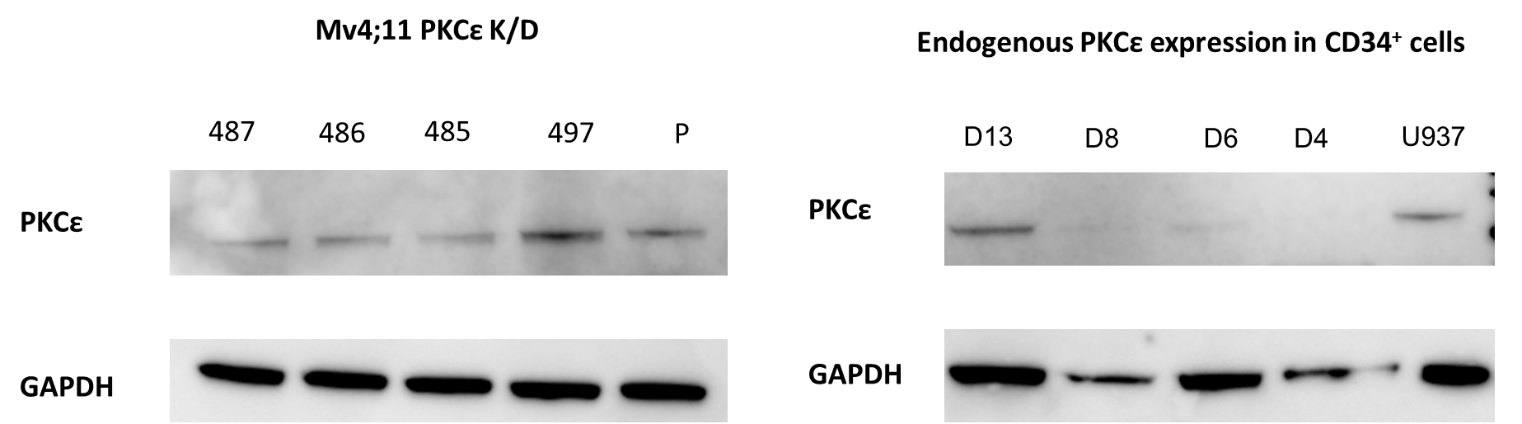

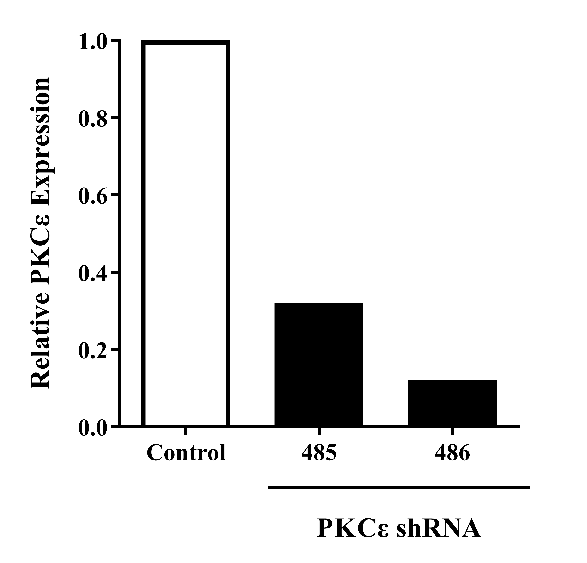

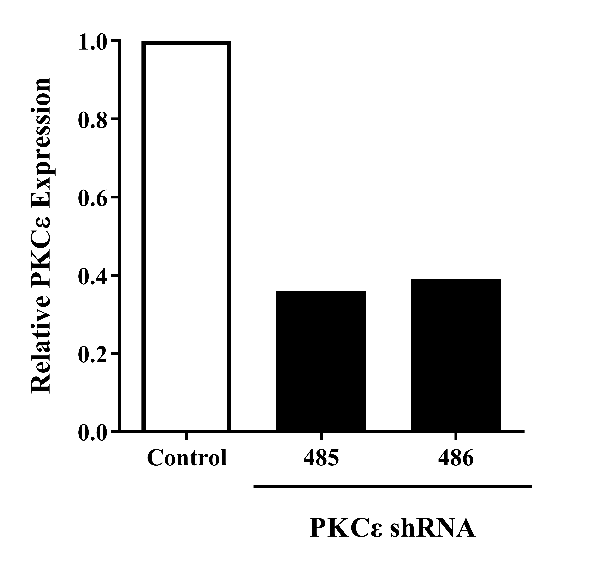


Western blot images (left) and quantification (right) showing PKCε (MW-84kDa) expression in **(A)** U937 and **(B)** Mv4;11 cells transduced control (shRNA_GFP_puro^R^ with no mammalian target; Supplementary Methods) and PKCε-targeted shRNA constructs (PKCε_shRNA_GFP_Puro^R^; 485 and 486; Supplementary Methods). Before lysate generation, the transduced cell lines underwent puromycin selection to remove un-transduced cells (See Materials and methods). Parental U937 and Mv4;11 cells were included in the western blot analysis to shown endogenous PKCε expression. PKCε expression was detected using the Cell Signaling Technologies antibody (22B10; Supplementary Table 3) and is shown alongside GAPDH (MW-36kDa) expression, which was used as a loading control and detected using the ThermoFisher Scientific GAPDH antibody (GA1R; Supplementary Table 3). PKCε expression was quantified by densitometry analysis using Image J (Fiji) as described in the Materials and Methods. PKCε expression was calculated relative to loading (GAPDH expression) and normalised to the expression of cells transduced with the control shRNA construct; n=1.

**A**

**B**

**U937**

**Mv4;11**

**PKCε shRNA**

**PKCε shRNA**

**PKCε shRNA**

**PKCε shRNA**

## Supplementary Figure 18. PKCε knockdown does not affect the growth or viability of AML cell lines

Bar charts representing the fold expansion (right) and viability (%; left) of **(A)** U937 and **(B)** Mv4;11 cells transduced with the control or PKCε-targeted shRNA constructs, described in Supplementary Methods, following 48 hours of culture. The fold expansion and viability of the cells was determined by TOPRO-3 (see Materials and methods); data represents mean+1SD; U937 (n=3), Mv4;11 (n=2). Statistical significance was determined using a one-way ANOVA with Bonferroni post‑test comparison and the data was deemed non-significant.


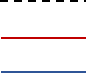

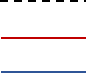


**EC50**

**Control:** 83nM±34nM

**485:** 125nM±94nM

**486:** 59nM±21nM

**EC50**

**Control:** 22nM±7nM

**485:** 20nM±6nM

**486:** 23nM±3nM

**A**

**B**

**Ara-C**

**DNR**

**EC50**

**Control:** 275nM±163nM

**485:** 427nM±311M

**486:** 235nM±129nM


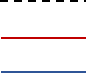

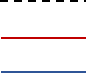


**EC50**

**Control:** >800nM

**485:** >800nM

**486:** 800nM

**U937**

**Mv4;11**

**U937**

**Mv4;11**

## Supplementary Figure 19: PKCε knockdown has no effect on Ara-C or DNR sensitivity of AML cell lines

Line graphs showing the effect of increasing **(A)** Ara-C and **(B)** DNR concentration (nM) on the viability of U937 and Mv4;11 cells transduced with the control or PKCε-targeted shRNA constructs described in Supplementary Methods, following 48 hours of compound treatment. Viability was measured by flow cytometry as described in the Materials and methods and normalised to cells treated with the vehicle control; data represents mean ± 1SD, n=2.


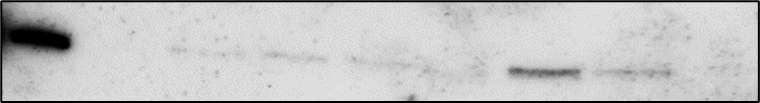


**P-GP**

**KG-1**

**148**

**160**

**162**

**164**

**165**

**172**

**177**

**92**

**AML patient-derived samples**


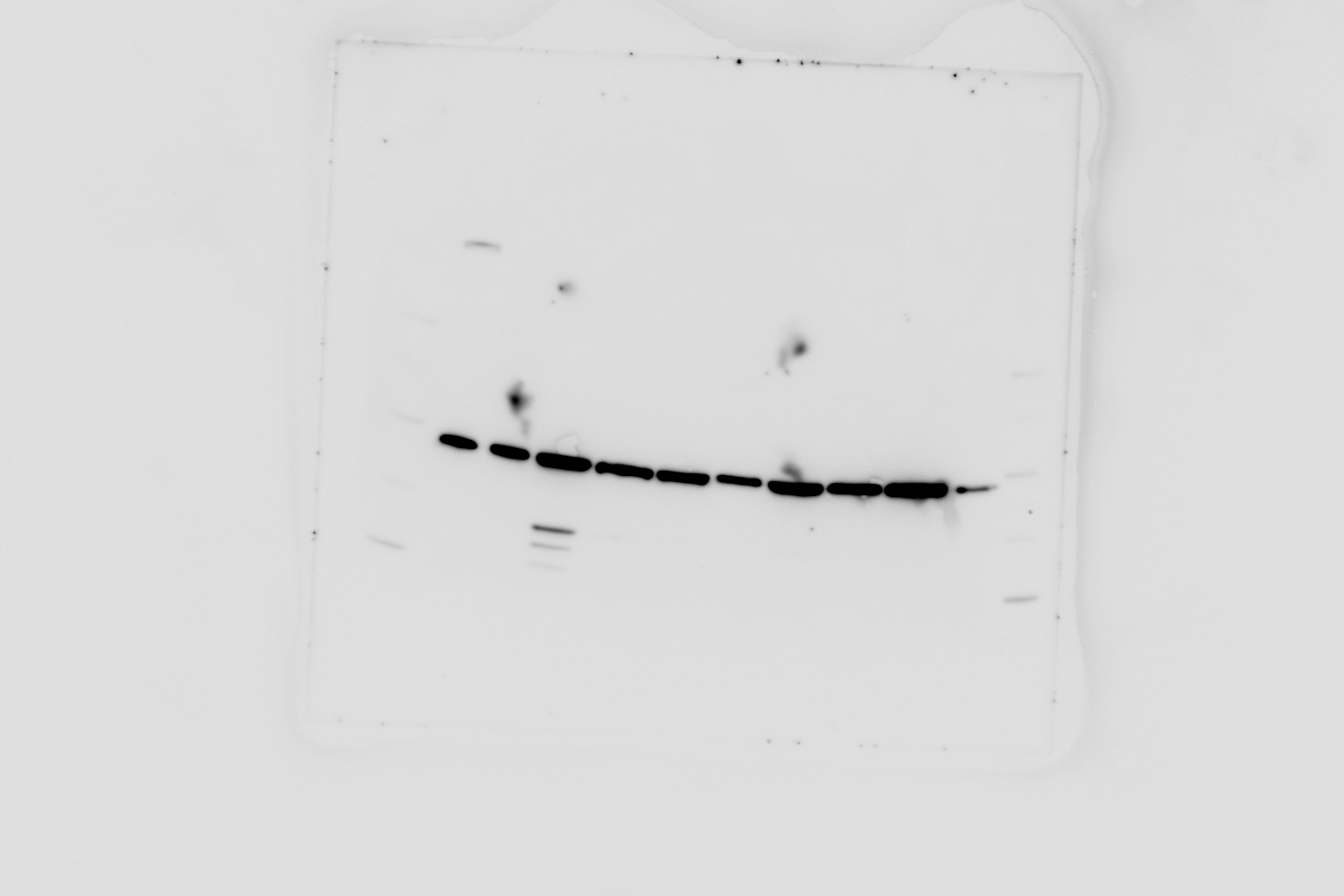


**PKCε**

**GAPDH**

**92**

**Mv4;11**

**148**

**160**

**162**

**164**

**165**

**172**

**177**

**AML patient-derived samples**

**GAPDH**


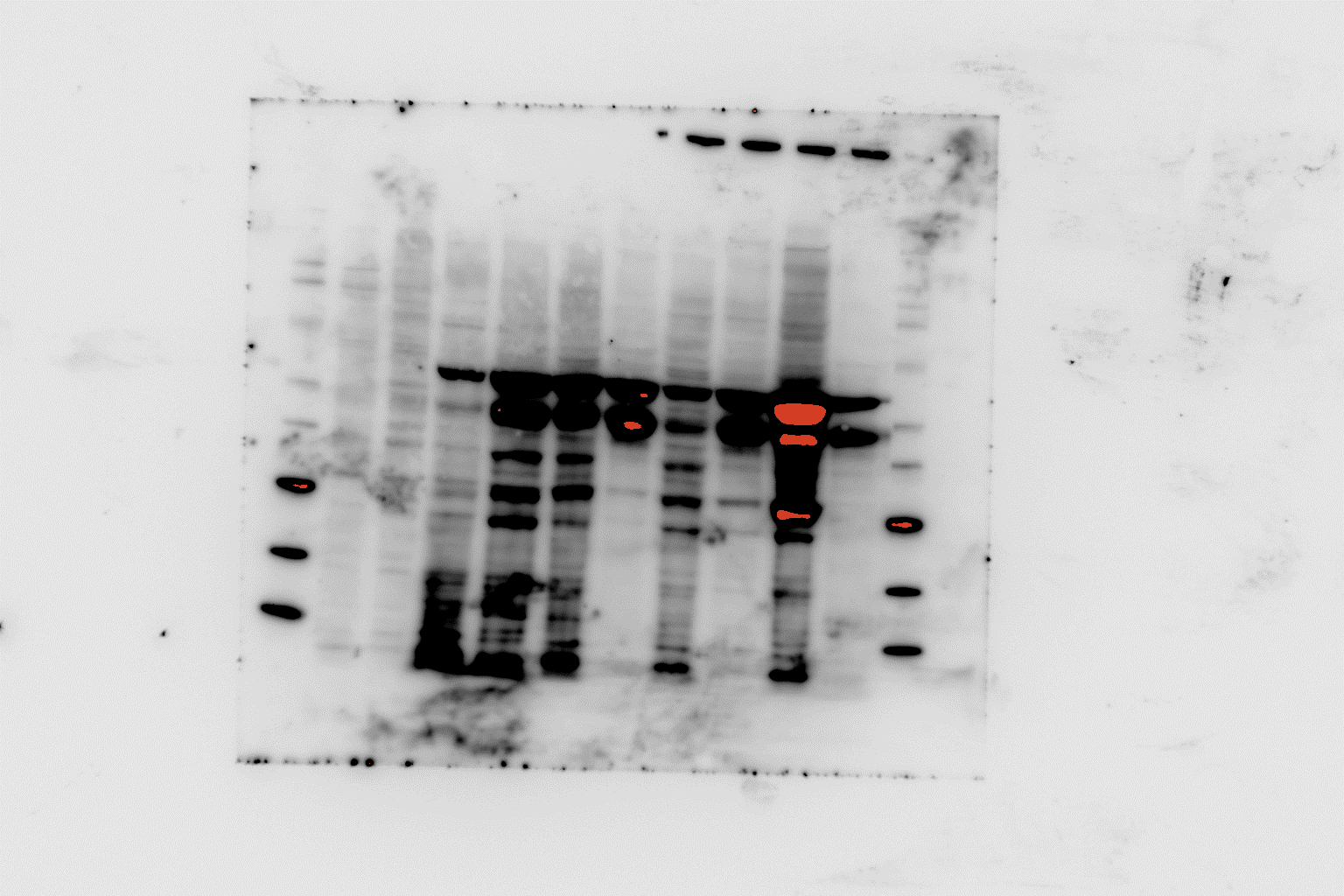


**A**

**B**


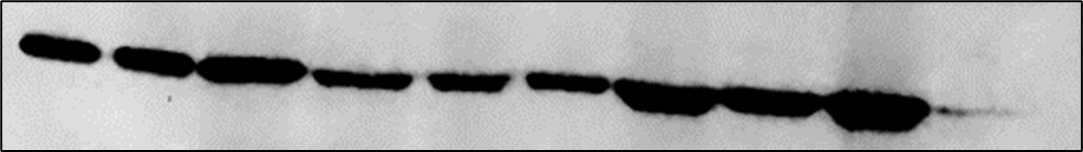


## Supplementary Figure 20. PKCε and P-GP protein expression in AML patient-derived samples

Western blot showing the expression of **(A)** PKCε (MW-84kDa) and **(B)** P-GP (MW140‑170kDa) expression in AML patient-derived samples (Supplementary Table 4). . PKCε expression was detected using the Cell Signaling Technologies antibody (22B10; Supplementary Table 3) while P-GP was detected using the Insight antibody (C219; Supplementary Figure 3). PKCε and P-GP are shown alongside GAPDH (MW-36kDa) expression, which was used as a loading control and detected using the ThermoFisher Scientific antibody (GA1R; Supplementary Table 3).

## Supplementary Figure 21. PKCε knockdown has no impact on DNR accumulation in AML patient samples

Bar chart showing the DNR fluorescence in the presence and absence of the P-GP inhibitor ZSQ (100nM), following 2 hours of treatment with 100nM DNR in AML patient samples; **(A)** AML165 and **(B)** AML172 transduced with the control or PKCε knockdown (486) constructs (Supplementary Methods). DNR fluorescence was normalised to the fluorescence of untreated treated with the vehicle controls (PBS or PBS+0.025% (*v/v*) DMSO); DNR n=3. DNR+ZSQ n=1; data represents mean+1SD. Statistical significance was determined using a Mann-Whitney test and was deemed non-significant.

## Supplementary Tables

## Supplementary Table 1. AML14 and AML15 Patient Samples Information

Table outlining the patient characteristics including gender, age, and AML disease classification (where available) for the AML14 and AML15 clinical trial samples analysed in this study.

| **Sample** | **Gender** | **Age (Years)** | **Cytogenetics** | **FAB** |
| --- | --- | --- | --- | --- |
| **1** | Male | 58 | N/A | N/A |
| **2** | Male | 47 | N/A | N/A |
| **3** | Male | 39 | Normal | M4 |
| **4** | Female | 54 | N/A | M4 |
| **5** | Male | 25 | inv(16) | M4 |
| **6** | Male | 25 | N/A | M4 |
| **7** | Female | 52 | Normal | N/A |
| **8** | Male | 44 | Normal | M5 |
| **9** | Female | 43 | N/A | M4 |
| **10** | Male | 33 | N/A | M5 |
| **11** | Female | 55 | N/A | M1 |
| **12** | Female | 39 | N/A | M4 |
| **13** | Female | 48 | Normal | M4 |
| **14** | Female | 51 | inv(16) | M4 |
| **15** | Female | 25 | N/a | M5 |
| **16** | Female | 58 | Normal | M2 |
| **17** | Male | 59 | N/A | N/A |
| **18** | Male | 37 | N/A | M6 |
| **19** | Female | 24 | Normal | M5 |
| **20** | Female | 19 | Other | Biphenotypic |
| **21** | Male | 53 | Normal | M5 |
| **22** | Female | 62 | N/A | N/A |
| **23** | Female | 18 | N/A | N/A |
| **24** | Female | 25 | N/A | M1 |
| **25** | Male | 58 | normal | M5 |
| **26** | Female | 56 | Normal | M5b |
| **27** | Male | 54 | Normal | M4 |
| **28** | Female | 50 | N/A | M1 |
| **29** | Female | 56 | N/A | M1 |
| **30** | Male | 61 | N/A | M5 |
| **31** | Female | 38 | N/A | N/A |
| **32** | Male | 17 | N/A | M1 |
| **33** | Male | 22 | Other | M1 |
| **34** | Male | 32 | Normal | M0 |
| **35** | Male | 56 | Normal | M1 |
| **36** | Male | 56 | N/A | M4 |
| **37** | Male | 55 | N/A | M1 |
| **38** | Male | 41 | N/A | N/A |
| **39** | Female | 33 | t(8;21) | M2 |
| **40** | Male | 60 | Complex | M1 |
| **41** | Male | 55 | N/A | M2 |
| **42** | Female | 48 | Normal | M2 |
| **43** | Female | 54 | N/A | N/A |
| **44** | Female | 56 | N/A | M4 |
| **45** | Female | 42 | N/A | M1 |
| **46** | Female | 42 | N/A | M1 |
| **47** | Female | 46 | N/A | M5 |
| **48** | Male | 64 | N/A | M1 |
| **49** | Male | 35 | N/A | M2 |
| **50** | Male | 24 | N/A | M4 |
| **51** | Male | 17 | N/A | M2 |
| **52** | Female | 56 | N/A | M1 |
| **53** | Male | 29 | N/A | M2 |
| **54** | Female | 42 | N/A | M2 |
| **55** | Female | 45 | N/A | M1 |
| **56** | Male | 48 | N/A | M1 |
| **57** | Male | 42 | N/A | M2 |
| **58** | Female | 34 | N/A | M5 |
| **59** | Female | 34 | N/A | M5 |
| **60** | Female | 53 | N/A | M0 |
| **61** | Male | 78 | Other | M2 |
| **62** | N/A | 1 | N/A | Mo |
| **63** | Female | 59 | t(8;21) | N/A |
| **64** | N/A | 2 | N/A | M1 |
| **65** | N/A | N/A | N/A | R |
| **66** | N/A | 24 | N/A | M1 |
| **67** | N/A | N/A | N/A | N/A |
| **68** | N/A | N/A | N/A | N/A |
| **69** | N/A | N/A | N/A | N/A |
| **70** | Male | 1 | N/A | N/A |
| **71** | male | 56 | Normal | N/A |
| **72** | Female | 56 | Normal | M5b |
| **73** | Male | 48 | inv(16) | M4e |
| **74** | Male | 25 | t(8;21) | M1 |
| **75** | N/A | 71 | Normal | M1 |
| **76** | Male | 65 | Normal | M1 |
| **77** | male | 62 | Normal | M5 |

## Supplementary Table 2. Summary of antibodies used for flow cytometric and western blot analysis

Table outlining the antibodies used for flow cytometric and western blot analysis in this study including antibody clone, conjugate, isotype control, concentration, manufacturer and catalogue number.

| **Flow Cytometry Antibodies** | | | | | | |
| --- | --- | --- | --- | --- | --- | --- |
| **Antibody** | **Conjugate** | **Clone** | **Isotype** | **Concentration** | **Manufacturer** | **Cat#** |
| IgG1 | PE | - | - | 5 ng/µL | BioLegend^®^ | 400140 |
| IgG2ka | APC | - | - | 5 ng/µL | BioLegend^®^ |  |
| CD11b | PE | ICRF4 | IgG1 | 5 ng/µL | BioLegend^®^ | 301306 |
| CD13 | APC | WM15 | IgG | 5 ng/µL | BioLegend^®^ | 301706 |
| CD14 | PE | HCD14 | IgG1 | 5 ng/µL | BioLegend^®^ | 325606 |
| CD15 | PE | W6D3 | IgG1 | 5 ng/µL | BioLegend^®^ | 325006 |
| CD34 | PE | 581 | IgG1 | 5 ng/µL | BioLegend^®^ | 345802 |
| CD36 | Biotinylated | SMO | - | 1 ng/µL | Ancell Corporation | 185-030 |
| CD243 | APC | UIC2 | IgG2ka | 5 ng/µL | BioLegend^®^ |  |
| Streptavidin | PerCP | - | - | 5 ng/µL | BD Pharmingen | 551419 |
| **Western Blot Antibodies** | | | | | | |
| **Antibody** | **Conjugate** | **Clone** | **Species** | **Dilution** | **Manufacturer** | **Cat#** |
| PKCε | - | 22B10 | Rabbit | 1:1000-1:5000 | Cell Signaling | 2683 |
| P-GP | - | C219 | Mouse | 1:5000 | Insight Biotechnology | GTX23364 |
| GAPDH | - | 6c5 | Mouse | 1:5000 | Santa Cruz | sc-32233 |
| GAPDH | DyLight (680nm) | GAIR | Mouse | 1:5000 | ThermoFisher Scientific | MA5-15738-D680 |
| Anti-Mouse Secondary | HRP | Polyclonal | Donkey | 1:5000 | GE HealthCare Scientific | NA931 |
| Anti-Rabbit Secondary | HRP | Polyclonal | Donkey | 1:5000 | GE HealthCare Scientific | NA934 |

## Supplementary Table 3. Agents used in drug sensitivity assays

Table outlining the agents, concentrations and vehicle controls used in drug sensitivity assays. *Abbreviations: Ara-C; cytarabine, ATM; antimycin A, ATO; arsenic trioxide, DNR; daunorubicin, GOx; glucose oxidase, PMA; Phorbol 12-myristate 13-acetate; ZSQ; zosuquidar hydrochloride.*^1^ Concentrations refer to the diluent, when used as a vehicle control these were diluted at 1µL/100µL of culture.

| **Drug** | **Manufacturer** | **Preparation of Master and working stocks** | **Cat no.** | **Diluent/ vehicle control^1^** | **Concentration** |
| --- | --- | --- | --- | --- | --- |
| Ara-C | Sigma-Aldrich | A 119mM Ara-C stock was generated by dissolving 100 mg solid Ara-C in 3 mL PBS. From this 840 µL was diluted in 9.2 mL PBS to generate a 10 mM working solution. | C1768 | PBS | 0-800nM |
| ATM | Sigma-Aldrich | A 20 Mm solution was generated by adding 2.3 mL ethanol to 25 mg ATM. From this a 2 mM working stock was generated by adding 100 μL ATM (20 mM stock) to 900 μL DMSO using a positive displacement pipette. | A8674 | DMSO | 0-20µM |
| ATO | Sigma-Aldrich | A 5 mM stock was generated by dissolving c20mg of solid ATO in 31 mL tissue-culture grade water (0.65 mg/mL). This was passed through a 0.22 µm filter and stored at RT. | 71287 | PBS | 0-4µM |
| DNR | Cayman Chemical | A 20mM master stock was generated by dissolving 5mg of solid DNR in 443µL DMSO. | 14159 | PBS | 0-100nM |
| GOx | Sigma-Aldrich | GOx (specific activity 192U/mg) was solubilised at 1mg/mL (equivalent to 192,000mU/mL) in PBS. When thawed for use, aliquots were used once. | 49180 | PBS+1% (*w/v*) BSA | 0-100mU |
| PMA | Cayman Chemical | A 100μM master stock was prepared in PBS and subsequently diluted 1/10 in PBS to generate a 10μM working stock. | 400145 | PBS | 1μM |
| ZSQ | Cayman Chemical | A 1mM stock was generated by dissolving 1.57mL DMSO. This 1mM stock solution was further diluted 1/100 in DMSO to make a 10µM working stock. | 21533 | PBS+2.5% (*v/v*) DMSO | 100nM |

## Supplementary Table 4: Description of AML patient-derived cell lines

Table outlining the patient characteristics of the AML patient-derived cell lines, patient age, and gender, the FAB and karyotypic characteristic of the disease, and the survival and relapse status of each patient if known. Fields where the clinical data is unknown is represented by a dash (-). *Abbreviations and definitions: CR; complete remission, censored; missing data, del; deletion; idem;* *Isoderivative chromosome, IND;induction.*

| **Cell line** | **Gender** | **Age** | **FAB** | **Karyotype** | **Survival** | **Relapse** |
| --- | --- | --- | --- | --- | --- | --- |
| AML92 | - | - | - | - | - | - |
| AML148 | Male | 46 | M4 | 46, XY, del (9)  (q13q22.3)  [4]/46, XY [17] | Alive | CR |
| AML160 | Female | 26 | M4 | 46, XX [20] | Deceased | CR |
| AML162 | Male | 40 | - | 46, XY [20] | Deceased | IND DEATH |
| AML164 | Female | 56 | M4 | 46, XX, del (7)  (q32q36)  [3]/47, idem, +14[7] | Deceased | CR |
| AML165 | - | - | - | - | - | - |
| AML172 | Female | 52 | M1 | 46, XX [20] | Alive | CENSORED |
| AML177 | - | - | - | - | - | - |

## References

1. Ley TJ, Miller C, Ding L, Raphael BJ, Mungall AJ, Robertson A, et al. Genomic and epigenomic landscapes of adult de novo acute myeloid leukemia. N Engl J Med. 2013;368(22):2059-74.

2. Rapin N, Bagger FO, Jendholm J, Mora-Jensen H, Krogh A, Kohlmann A, et al. Comparing cancer vs normal gene expression profiles identifies new disease entities and common transcriptional programs in AML patients. Blood. 2014;123(6):894-904.

3. Di Tullio A, Vu Manh TP, Schubert A, Castellano G, Månsson R, Graf T. CCAAT/enhancer binding protein alpha (C/EBP(alpha))-induced transdifferentiation of pre-B cells into macrophages involves no overt retrodifferentiation. Proc Natl Acad Sci U S A. 2011;108(41):17016-21.

4. Chambers SM, Boles NC, Lin KY, Tierney MP, Bowman TV, Bradfute SB, et al. Hematopoietic fingerprints: an expression database of stem cells and their progeny. Cell Stem Cell. 2007;1(5):578-91.

5. Berg JS, Lin KK, Sonnet C, Boles NC, Weksberg DC, Nguyen H, et al. Imprinted genes that regulate early mammalian growth are coexpressed in somatic stem cells. PLoS One. 2011;6(10):e26410.

6. Bagger FO, Sasivarevic D, Sohi SH, Laursen LG, Pundhir S, Sonderby CK, et al. BloodSpot: a database of gene expression profiles and transcriptional programs for healthy and malignant haematopoiesis. Nucleic Acids Res. 2016;44(D1):D917-24.

7. Yang X, Boehm JS, Yang X, Salehi-Ashtiani K, Hao T, Shen Y, et al. A public genome-scale lentiviral expression library of human ORFs. Nat Methods. 2011;8(8):659-61.
